# Patterns of Avian Frugivory in Beijing's Urban Forest Across Multiple Temporal Scales

**DOI:** 10.1002/ece3.72699

**Published:** 2025-12-17

**Authors:** Xinyi Liu, Xudong Yang, Jing Zhou, Xinyu Li, Zhitao Guo, Jun Yang

**Affiliations:** ^1^ Department of Earth System Science, Ministry of Education Ecological Field Station for East Asian Migratory Birds, Institute for Global Change Studies Tsinghua University Beijing China; ^2^ Beijing Key Laboratory of Ecological Function Assessment and Regulation Technology of Green Space, Beijing Urban Ecosystem National Observation and Research Station Beijing Academy of Forestry and Landscape Architecture Beijing China; ^3^ Beijing Rujing Ecological Landscaping Co., Ltd. Beijing China

**Keywords:** arboreal camera trapping, frugivory, human disturbance, temporal scale, urban environment

## Abstract

Avian frugivory is a vital process shaping the diversity of bird and plant species. Nevertheless, how this process varies temporally in urban forests is not well understood. In this study, we employed arboreal camera trapping to investigate the temporal patterns of bird–fruiting tree interactions across hourly to annual time scales in three urban forest sites in Beijing, China. We captured 618 independent frugivory events involving 19 bird species and 12 fruiting tree species from 584,886 images recorded in 12 months. Large‐scale temporal events like fruiting phenology and bird migration had primary effects on the seasonal patterns of frugivory events, while site conditions, including fruit tree diversity and human activities, significantly affected the monthly and diurnal patterns. The structure of the interaction network also reflected the effects of these factors. Our results show that maintaining consistent fruit availability and minimizing human disturbances are critical for sustaining avian frugivory in urban forests. Additionally, the results show that observations at high temporal resolutions are indispensable for understanding avian frugivory in urban environments.

## Graphical abstract

Based on 584,886 arboreal camera trap photos collected over 12 months, we identified 618 frugivory events across 19 bird species and 12 fruiting tree species in Beijing's urban forests. Fruiting phenology, bird migration, and human disturbance strongly influenced temporal patterns of avian frugivory, highlighting strategies for sustaining biodiversity in urban ecosystems.

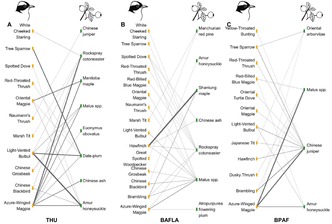

## 1 Introduction

Birds are a key component of the urban ecosystem and a sensitive indicator of ecological change (La Sorte et al. 2018; Sidemo‐Holm et al. 2022; Sol et al. 2017, 2020). Therefore, considerable efforts have been devoted to preserving bird diversity in urban environments. Urban bird diversity is influenced by various environmental and anthropogenic factors, with food availability playing a significant role (Ciach and Fröhlich 2017; Galbraith et al. 2015; Patankar et al. 2021). Food directly affects bird species richness and distribution, making it a key factor in sustaining species interactions and ecosystem functioning in human‐dominated landscapes (Marcacci et al. 2021).

In urban environments, fruiting trees provide critical food resources for frugivorous birds, particularly during periods of food scarcity, such as winter and early spring (Andrade et al. 2011; He et al. 2021; Zhang et al. 2022). Previous studies have shown significant associations between fruiting plants and frugivorous birds in urban environments. Zietsman et al. (2019) found that the Rufous‐bellied thrush ( *Turdus rufiventris* ) moved between native and artificial habitats depending on fruit availability. Liu et al. (2025) showed that bird and fruit tree richness were positively correlated across 24 urban parks. Li et al. (2025) identified fruit abundance and plant structural traits, such as the height of woody plants and the density of branches and leaves, as key predictors of bird richness and abundance in urban environments, consistent with findings from other studies (Ramos‐Robles et al. 2016; Wang et al. 2023). These studies demonstrate that avian frugivory, a key interspecific interaction that shapes patterns of frugivorous bird diversity and community structure in natural environments (Case and Tarwater 2020; Ferger et al. 2014; Kissling et al. 2007), is also influencing the richness and composition of frugivorous birds in urban environments.

Although studies on avian frugivory in urban environments are increasing, the temporal dynamics of interactions between birds and fruiting trees remain poorly understood. Most observational studies have identified a seasonal pattern, that is, avian frugivory intensifies in seasons when other food sources are scarce (Yin et al. 2023; Yong et al. 2014). However, how the interaction varies at finer temporal scales is not known. Finer temporal scales uncover subtle differences in bird foraging behavior, such as seasonal changes in feeding activity, which are essential for understanding how resources are used in urban habitats. CaraDonna et al. (2021) demonstrated that frugivory is highly variable at fine temporal scales, with notable differences in interaction patterns. Likewise, Gleditsch et al. (2017) found that resource tracking by frugivores results in temporal fluctuations in foraging intensity. Lack of data at the finer temporal scales is the main cause. Existing studies relied on field surveys adopting transect or point sampling methods (Khan et al. 2023; Li et al. 2025), which are labor‐intensive and time‐consuming. Therefore, observers often choose to investigate a site once or twice each month at a specific time of day, such as one hour after sunrise, to capture a glimpse of avian frugivory (Kim et al. 2016). The sparse observation data limit the observers' ability to detect fine‐scale patterns, such as daily or hourly foraging rhythms (Pais De Faria et al. 2021). The lack of understanding of the temporal variation of avian frugivory becomes a barrier to taking conservative actions to maintain the urban birds' diversity and ecosystem functions.

Besides adopting a coarse temporal resolution, existing studies on avian frugivory in urban environments often focus on frugivorous birds and fruit trees (Khan et al. 2023; K. Li et al. 2025; Liu et al. 2025). The influence of human activity on avian frugivory has rarely been considered, although some studies found that increased human disturbance reduces the richness of frugivorous birds and promotes the dominance of generalists in urban areas (Gutiérrez‐Tapia et al. 2018; Kale et al. 2018; Ramaswami et al. 2016). Additionally, some studies have shown that anthropogenic disturbance can influence the network of frugivorous birds and fruiting trees, such as simplifying the interaction (Schneiberg et al. 2020; Zhang et al. 2022). Nevertheless, the effects of human activities on the timing and structure of bird–fruiting tree interactions in urban environments remain understudied. Human activities affect bird behavior by changing feeding times and increasing variability in foraging patterns. Pejchar et al. (2008) demonstrated that human disturbance can disrupt bird behavior and seed dispersal, while Zietsman et al. (2019) showed that human disturbances simplify the bird–fruit interaction network, impacting frugivore visitation and seed dispersal. The sparse observation data is again a barrier to statistical analysis and interpretation. The above limitations prevent us from answering questions like: When are bird–fruiting tree interactions most intense in urban environments across a day, a month, or a year? Which bird or tree species is playing a dominant role at those time scales? The answers to the questions are essential for guiding the conservation of frugivorous birds in urban areas.

With the above limitations in mind, this study aims to address the research question: How does avian frugivory vary across temporal scales in an urban environment? To answer this question, we employed arboreal infrared camera traps and an AI‐based image analysis tool to record and analyze frugivory events with high temporal resolution at urban sites. Arboreal infrared camera traps have been used in natural environments to capture the long‐term dynamics of avian frugivory at a high temporal resolution (Zhu et al. 2022). Nevertheless, their use in urban environments for the same purpose was still rare (Raji and Downs 2022). Combining this approach with a network perspective further reveals how bird–tree interactions are organized and how urban frugivorous bird communities respond to disturbance (Gilarranz et al. 2017; Thébault and Fontaine 2010). It is known that network metrics, such as nestedness, can indicate effective interspecific competition, coexistence, and stability (Bastolla et al. 2009). Modularity reflects compartmentalization that can localize perturbations and buffer community‐wide impacts (Gilarranz et al. 2017). Interaction diversity and interaction evenness summarize how interaction frequencies are distributed across species and inform specialization, partner sharing, and functional redundancy in urban systems (Blüthgen et al. 2006). Our study addresses the following three specific objectives: (1) to reveal the temporal pattern of frugivory events across different sites; (2) to examine the compositional change of bird species, tree species, and interaction pairs; and (3) to assess the temporal change of the bird–fruiting tree interaction network's structure at different sites.

## 2 Materials and Methods

### 2.1 Study Area

**FIGURE 1:**
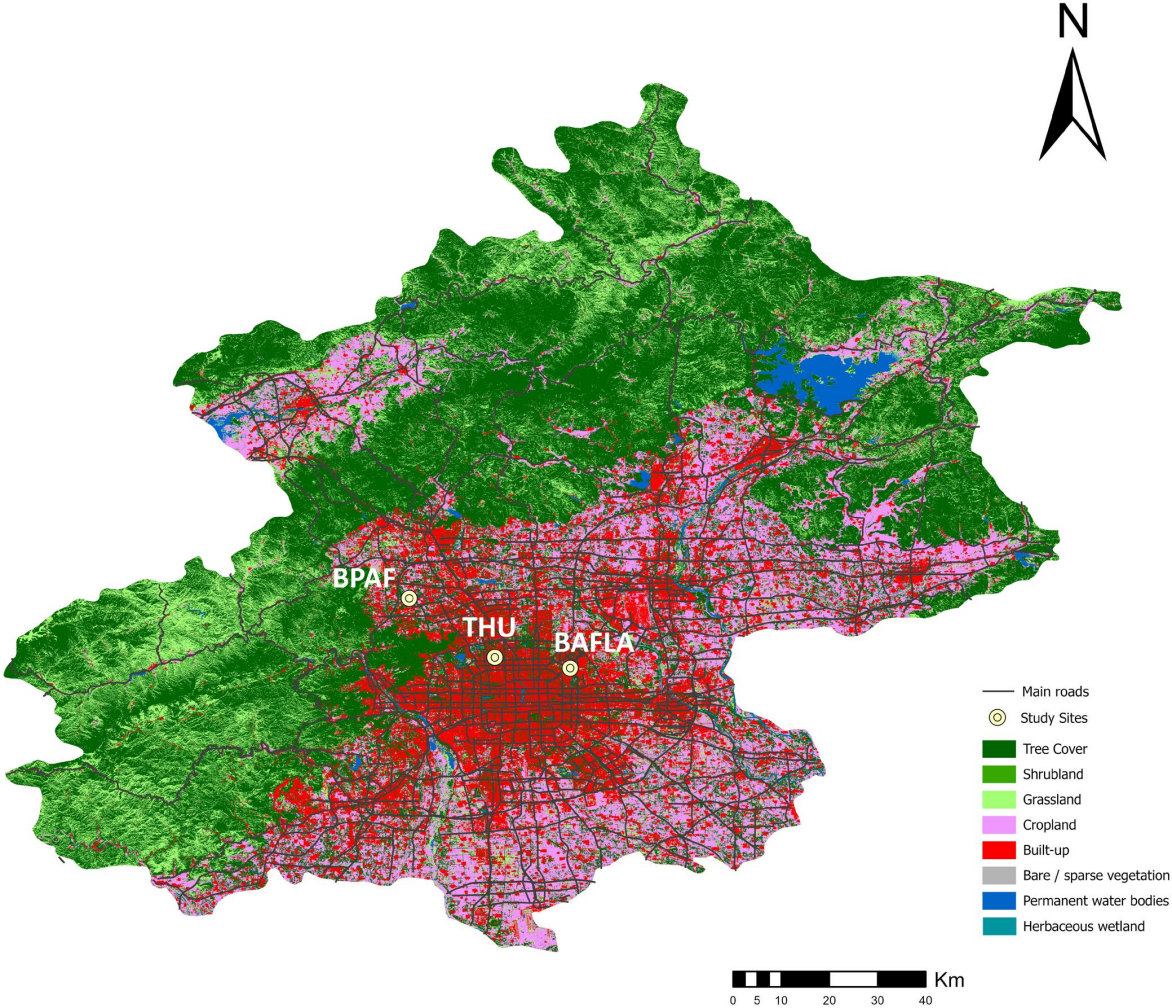
The map of the study sites: Tsinghua University (THU), Beijing Academy of Forestry and Landscape Architecture (BAFLA), and Beijing Plain Area Forest (BPAF). The sites are depicted on Beijing's land cover map in 2022, with main roads included for reference.

Beijing, China (115°42′–117°24′ E, 39°24′–41°36′ N) is located in the warm‐temperate sub‐humid region (Zhao 2019). Three sites in Beijing's urban forest were chosen for this study: Tsinghua University (THU), Beijing Academy of Forestry and Landscape Architecture (BAFLA), and a planting site of Beijing Plain Area Forest (BPAF) (Figure 1). The 483‐ha THU campus hosts around 80,000 students, faculty members, and staff, representing a high human activity site. BPAF, as part of a 133‐ha tree plantation located in suburban Beijing, is subjected to low‐intensity management and low human activities. BAFLA is a research institute with around 180 staff members working on 13 ha of land. The human activity level on the ground falls between those at THU and BPAF.

### 2.2 Data Collection

A field survey was initially conducted at each site to identify fruiting trees for camera installation. The monitoring took place from December 2023 to November 2024, during which memory cards and batteries were replaced regularly. The naiveness of each species was evaluated at the regional scale by referring to the *Flora of China* (Li 2007). Species naturally distributed in Beijing were considered native. Species without a natural distribution in Beijing, and planted exotics and cultivars, were treated as non‐native or cultivated. Once a fruiting tree was identified, its suitability for camera placement was evaluated based on the visibility and accessibility of fruit‐bearing branches. To minimize redundancy in data collection, cameras were installed at locations without overlapping fields of view and spaced at least 2–3 m apart when multiple fruiting individuals of the same species were nearby.

A total of 35 infrared digital cameras (LTL 6210MC, L710) were installed at the three sites (THU: 11; BAFLA: 12; BPAF: 12). The cameras were installed at a height of 0.5–8 m above the ground, pointing toward target branches with high fruit densities to maximize the detection probabilities of visiting frugivorous birds. Cameras were installed within continuous green space patches at each site, avoiding areas of heavy human activity such as main paths or student zones. The cameras were configured to operate continuously and record three photos, followed by a 10‐s video, upon triggering. A 10‐s delay was implemented between triggers to mitigate the rapid consumption of memory cards when animals remained within the camera's field of view (Li et al. 2022; Zhu et al. 2022). The cameras automatically stamp the date and time on captured photos and videos. Tree species monitored with camera traps included: Amur honeysuckle, Atropurpurea flowering plum, Chinese ash, Chinese juniper, Date‐plum, *Euonymus obovatus* , *Malus* spp., Manitoba maple, Rockspray cotoneaster, and Shantung maple at THU; Amur honeysuckle, Atropurpurea flowering plum, Chinese ash, *Malus* spp., Manchurian red pine, Rockspray cotoneaster, and Shantung maple at BAFLA; and Amur honeysuckle, Chinese juniper, Chinese ash, *Malus* spp., and Oriental arborvitae at BPAF.

### 2.3 Data Analysis

**FIGURE 2:**
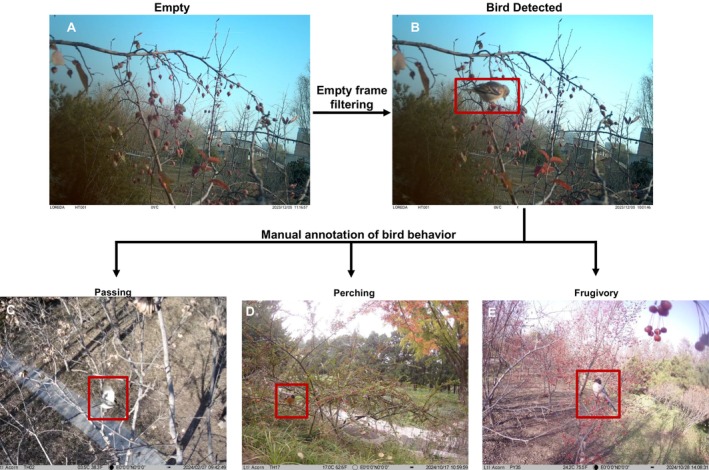
Workflow of image filtering and bird behavior annotation. (A) Image filtered as empty by MegaDetector. (B) Image detected as containing a bird. (C) A passing event where the bird flies through the frame without interaction. (D) A perching event where the bird lands but does not interact with the fruits. (E) A frugivory event where the bird pecks or swallows fruits. Only frugivory events were used in the analysis.

The images with birds were extracted from the recorded images and videos using a method that combines an AI‐based tool with manual verification (Figure 2A,B). MegaDetector is an AI‐based tool trained on millions of images from diverse ecosystems to detect animals, people, and vehicles in camera trap images (Beery 2023). It was used in this study to filter out empty images. To ensure the accuracy of the filtering process, 1% of filtered images from each monitoring site were randomly selected for manual validation. The recall rate of bird detections was used as the indicator of filtering accuracy (Equation 1). The filtering process was conducted iteratively until the recall rate was equal to or higher than 95%.

(1) $$\text{Recall}=\frac{\text{True Positives}}{\text{True Positives}+\text{False Negatives}}$$

In Equation (1), True Positives refer to the number of image files correctly identified as containing birds. In contrast, False Negatives refer to image files containing birds that were incorrectly classified as empty.

Annotation of bird behaviors was conducted manually using the Timelapse software package developed by Greenberg et al. (2019). The images with birds were classified into three categories: frugivory (Figure 2E), perching (Figure 2D), and passing (Figure 2C). Frugivory includes birds' fruit‐swallowing or pecking behaviors. Perching refers to birds' landing on branches, but no frugivory event was recorded. When birds appear within the camera frame without landing or engaging with the fruiting tree, it is classified as passing. Frugivory behaviors were further divided into independent frugivory events. Zhu et al. (2022) defined an independent interaction event as consecutive photos/videos of the same plant‐frugivore interaction separated by more than 5 min. To define independent frugivory events, we tested time gaps from 1 to 30 min and chose a 3‐min threshold based on sensitivity analysis (Table S10). Using this threshold, we identified 618 independent events. While Villalva et al. (2024) used a 5‐min interval in natural habitats, our more conservative threshold was better suited to capture the rapid visitation patterns in urban environments. A species' resident status in Beijing was determined by referring to Wild Birds of Beijing (Li 2014). In this study, migratory species are those that occur in Beijing seasonally. Resident species are those present year‐round in Beijing. Detailed resident status for each detected species is provided in Table S1. Bird taxonomy and English names used in this study were standardized following the IOC World Bird List v15.1 (Gill et al. 2025).

Seasonal, monthly, and daily variations in frugivory events were compared across the three sites. The four seasons in Beijing are spring (March–May), summer (June–August), autumn (September–November), and winter (December–February). Also, the occurrence of frugivory events at different times of day was examined. The number of independent frugivory events was summarized for each temporal scale. The significance of the location, time, and their interaction effects was tested using analysis of variance (ANOVA) based on permutation (Wheeler and Torchiano 2010).

At the annual, seasonal, and monthly levels, the difference in species composition of frugivorous birds for each location and time combination was examined using the Jaccard dissimilarity coefficient (JI). The frequency of bird species was counted to identify the dominant species at different locations and time combinations. To further examine the variation in interactions between birds and trees, the different bird–fruiting tree interaction pairs and their frequencies were used to calculate Bray–Curtis dissimilarity (BC) for each location and time combination. Variation at different times of the day was also examined. No comparison of species composition and interaction pairs was done at the daily sale because the daily variation was not the question addressed in this study.

To examine the characteristics of the interaction network formed by birds and trees, the bird–fruiting tree interaction networks were constructed at the annual and seasonal scales for each site. Four network metrics—weighted nestedness, modularity, interaction diversity, and interaction evenness—were calculated at both annual and monthly scales. High values of weighted nestedness indicate a tendency for species with few interactions to connect with highly connected species (Bastolla et al. 2009). Modularity measures the extent to which the network is organized into distinct modules (Barber 2007). Interaction diversity is based on the Shannon‐Wiener index and reflects variation in interaction frequency, while interaction evenness measures the uniformity in the distribution of interactions across species (Blüthgen et al. 2006; Dormann 2011). The values of the four metrics were compared to give a qualitative description of changes at different sites and seasons. Networks were not constructed for finer temporal scales due to the limited number of observations at those scales.

All analyses were conducted using R v.4.2.1 (R Core Team 2024). The Jaccard index and Bray‐Curtis index were calculated using the vegan package (Tikhonov et al. 2020). The ANOVA analysis was run using the lmPerm package (Wheeler and Torchiano 2010). The network analysis was implemented using the Bipartite package (Dormann 2011).

## 3 Results

### 3.1 Overall Patterns of Frugivore Events at the Three Sites

The infrared cameras captured 584,886 images and 195,058 video files. After filtering, 5586 pictures and 1862 videos were found to contain birds. The filtered pictures were classified into passing (107), perching (2685), and frugivore (2794) manually. Through further analysis, 618 independent frugivory events involving 19 bird species and 12 fruiting tree species (see Table S1 for a complete list of species names) were identified. Seven out of the 19 bird species were migratory and contributed to 190 of the 618 recorded frugivory events (30.7%). These included Yellow‐throated Bunting, Hawfinch, Chinese Grosbeak, Brambling, Naumann's Thrush, Red‐throated Thrush, and Dusky Thrush. Yellow‐throated Bunting primarily occurred in spring and autumn and was usually absent in mid‐winter. The other six migratory species were present throughout autumn and winter and contributed to frugivory events during these seasons (see Table S1). Five of the fruiting tree species do not have a natural distribution in Beijing. Also, it is difficult to classify the widely planted cultivars of *Malus* spp. into species, so they are treated as one taxon in this study. The remaining six tree species are all naturally distributed in Beijing.

**FIGURE 3:**
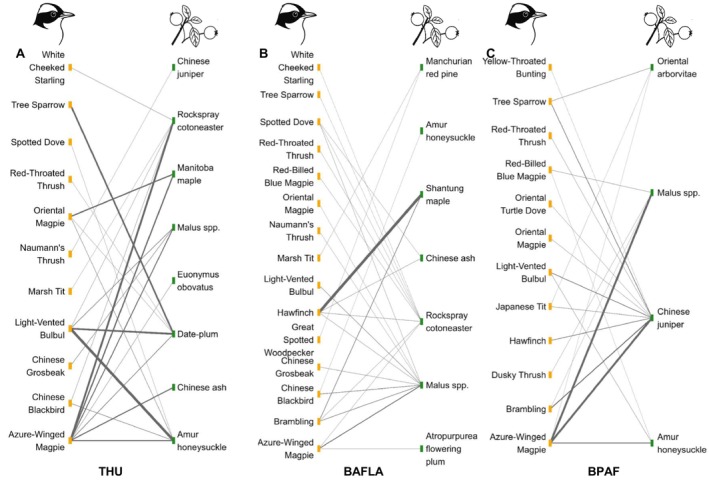
Bird‐fruiting tree interactions are shown for the three study sites: (A) Tsinghua University; (B) Beijing Academy of Forestry and Landscape Architecture; and (C) Beijing Plain Area Forest. In each panel, fruiting tree species are shown on the right, and bird species are shown on the left. Lines represent observed foraging interactions, with line thickness proportional to the frequency of interactions.

Across all sites, 58 unique bird–fruiting tree interaction pairs were documented (Figure 3). At BAFLA, 24 unique pairs involving 14 bird species and seven tree species were identified in 238 events. The number of unique pairs identified from 250 events at BPAF was 19, involving 12 bird species and four tree species. About 23 distinct pairs were identified in 130 events at THU, involving 11 bird species and eight tree species. The Azure‐winged Magpie displayed a wide foraging range among all bird species. *Malus* spp., Amur honeysuckle, Rockspray cotoneaster, and Chinese juniper experienced high frequencies of foraging interactions and were linked to more bird species than other tree species.

### 3.2 Temporal Variation in Frugivory Events Across Sites

**FIGURE 4:**
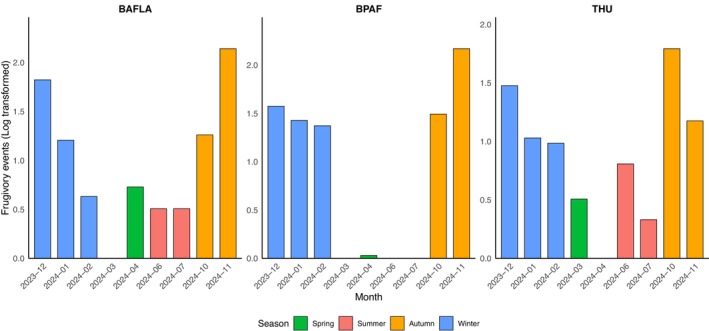
Seasonal and monthly variation in frugivory events (log‐transformed, a small value of 0.03 was added for display purposes as log_10_1 = 0) at THU, BAFLA, and BPAF from December 2023 to November 2024.

The number of frugivory events exhibited strong seasonal and monthly dynamics (Figure 4). The frugivory events peaked in the late autumn and lasted into the winter across all sites, while they were low and sporadic in the spring. ANOVA results indicated that the season had a significant effect, while the location and the interaction effect were not significant (Table 1).

**TABLE 1:** Analysis of variance table for frugivory events in different seasons and sites.

	df	*R* Sum Sq	*R* Mean Sq	Iter	Pr
Location	2	3700.7	1850.3	111	0.7477
Season	3	23620.9	7873.6	5000	0.0154*
Location:Season	5	3142.4	628.5	117	0.9915.
Residuals	0	0.0	NaN		

*Note:* Significance code: *p* < 0.001(***); *p* < 0.01(**); *p* < 0.05(*).

Monthly variation in frugivory events was largely the same at the three sites except in the summer. No event was detected at BPAF in May, June, or July. ANOVA results indicated that month, location, and the interaction effect all had a significant effect (Table S2).

**FIGURE 5:**
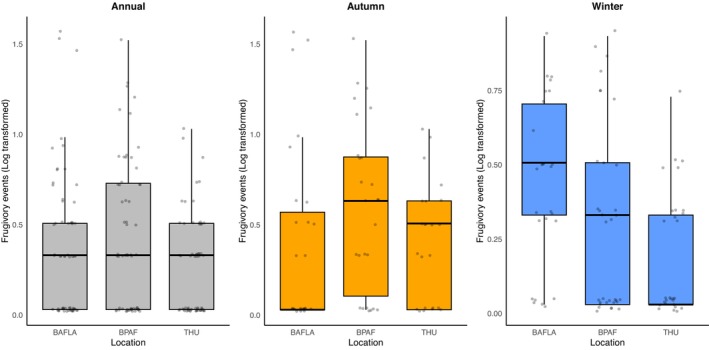
Daily variation in frugivory events (log‐transformed, a value of 0.03 was added for display purposes as log_10_1 = 0) at THU, BAFLA, and BPAF from December 2023 to December 2024.

Daily variation in frugivory events at THU was the smallest (Figure 5), followed by BPAF. Daily variation in the number of frugivory events at BAFLA was higher in winter than in autumn and throughout the entire year. ANOVA results indicated that the date, location, and their interaction effect had significant influences on daily frugivory events. All had Pr < 0.001 (Table S3).

**FIGURE 6:**
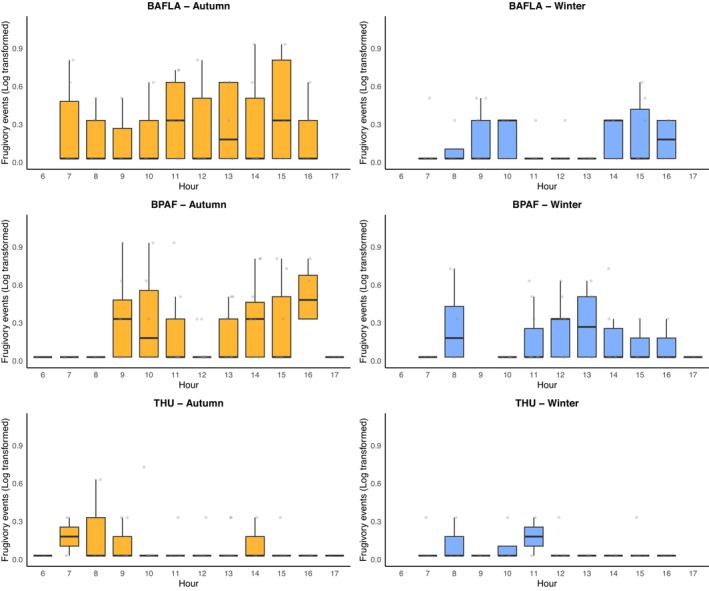
Hourly patterns of frugivory events (log‐transformed, a small value of 0.03 was added for display purposes as log_10_1 = 0) at THU, BAFLA, and BPAF during autumn and winter.

Frugivory events exclusively occurred during the daytime. ANOVA results indicated that the hour of the day and its interaction effect with location and season all had a significant influence on the number of frugivory events (Table S4). Because frugivory events in the autumn and winter accounted for a large portion of the observed data, only the hourly variation in two seasons was plotted here (Figure 6). In the autumn, frugivory events lasted throughout the day in BAFLA and BPAF. In THU, frugivory events occurred mainly from 7:00 to 9:00, then again around 14:00. Frugivory events for the rest of the day were at a low level. The difference was more obvious in the winter. Frugivory events in THU mainly occurred in the morning hours, while peaks in the morning and afternoon can be observed at BAFLA and BPAF.

### 3.3 Temporal Variation in Species Composition and Bird‐Fruiting Tree Pairs

At the annual scale, the species composition of birds involved in frugivory events at BAFLA was more similar to THU (JI = 0.33) than that of BPAF (JI = 0.56). BPAF and THU had the highest dissimilarity in species composition (JI = 0.72). Hawfinch was the most abundant species at BAFLA, with 227 individuals (16.8% of the total) recorded at this site. Azure‐winged Magpie was the most abundant species at BPAF and THU, with 479 individuals (35.5% of the total) and 72 individuals (5.3%) recorded at the two sites.

However, if evaluated using the composition of bird‐fruiting tree interaction pairs, the three sites were more dissimilar. The Bray–Curtis dissimilarity values were 0.89 between BAFLA and THU, 0.82 between BAFLA and BPAF, and 0.84 between BPAF and THU, respectively.

At the seasonal scale, the species composition of birds involved in the frugivory events varied among sites and seasons. Across sites, species in the same season were more similar than those in other seasons. Across seasons at the same site, those in the autumn and winter were more similar than in other seasons (Table 2).

**TABLE 2:** The dissimilarity of bird species composition at the three sites in different seasons measured in Jaccard dissimilarity coefficients (0 = identical sets; 1 = no similarity).

Location/season		BAFLA				BPAF			THU			
		Sp	Su	A	W	Sp	A	W	Sp	Su	A	W
BAFLA	Sp		0.00	0.88	0.93	1.00	0.75	0.90	0.00	0.67	0.83	0.83
	Su			0.88	0.93	1.00	0.75	0.90	0.00	0.67	0.83	0.83
	A				0.43	1.00	0.80	0.71	0.88	0.78	0.60	0.73
	W					0.93	0.80	0.59	0.93	0.79	0.67	0.57
BPAF	Sp						0.75	1.00	1.00	1.00	1.00	1.00
	A							0.83	0.75	0.83	0.75	0.75
	W								0.90	0.82	0.86	0.55
THU	Sp									0.67	0.83	0.83
	Su										0.88	0.71
	A											0.80
	W											

Abbreviations: A = Autumn, Sp = Spring, Su = Summer, W = Winter.

The composition of bird–fruiting tree interaction pairs revealed a seasonal pattern too (Table 3). For most combinations of sites and seasons, the dissimilarity levels were high. However, those in autumn and winter were more similar than in other seasons. The only exception was in BAFLA, where the composition of bird–fruiting tree interaction pairs was highly similar between spring and summer.

**TABLE 3:** The dissimilarity of bird–fruiting tree interaction pairs at the three sites in different seasons measured in Bray–Curtis dissimilarity index (0 = identical sets; 1 = no similarity).

Location/season		BAFLA				BPAF			THU			
		Sp	Su	A	W	Sp	A	W	Sp	Su	A	W
BAFLA	Sp		0.27	0.97	0.95	1.00	0.98	1.00	1.00	0.85	0.95	1.00
	Su			0.95	0.91	1.00	0.95	1.00	1.00	0.86	0.90	1.00
	A				0.62	1.00	0.88	1.00	1.00	0.99	0.86	1.00
	W					1.00	0.85	1.00	0.98	0.98	0.78	0.98
BPAF	Sp						1.00	1.00	1.00	1.00	1.00	1.00
	A							1.00	1.00	0.99	0.75	1.00
	W								1.00	1.00	1.00	1.00
THU	Sp									1.00	0.97	0.96
	Su										0.98	1.00
	A											0.98
	W											

Abbreviations: A = Autumn, Sp = Spring, Su = Summer, W = Winter.

Birds that visited fruiting trees varied among seasons at different sites (Table 4). However, the Azure‐winged Magpie had a dominant influence at the three sites. The bird–fruiting tree interaction pairs reflected this dominance. The tree species with the highest number of frugivory events was relatively consistent across sites, mainly including *Malus* spp., Amur honeysuckle, Rockspray cotoneaster, Shantung maple, Chinese ash, and Chinese juniper. They formed interaction pairs with the dominant bird species.

**TABLE 4:** The most abundant bird species and bird–fruiting tree interaction pairs in different seasons at the three sites.

Site	Season	Bird species	Count	%	Bird‐tree pair	Inc	%
BAFLA	Spring	Azure‐winged Magpie	5	0.4	Azure‐winged Magpie—Atropurpurea flowering plum	3	0.5
	Summer	Azure‐winged Magpie	7	0.5	Azure‐winged Magpie—*Malus* spp.	4	0.6
	Autumn	Hawfinch	210	15.6	Hawfinch—Shantung maple	62	10.0
	Winter	Brambling	69	5.1	Azure‐winged Magpie—*Malus* spp.	19	3.1
BPAF	Spring	Red‐billed Blue Magpie	1	0.1	Red‐billed Blue Magpie—Amur honeysuckle	1	0.2
	Autumn	Azure‐winged Magpie	404	29.9	Azure‐winged Magpie—*Malus* spp.	124	20.1
	Winter	Azure‐winged Magpie	75	5.6	Light‐wented Bulbul—Chinese juniper	21	3.4
THU	Spring	Azure‐winged Magpie	3	0.2	Azure‐winged Magpie—Rockspray cotoneaster	3	0.5
	Summer	Oriental Magpie	5	0.4	Oriental Magpie—Amur honeysuckle	5	0.9
	Autumn	Light‐vented Bulbul	46	3.4	Light‐vented Bulbul—Amur honeysuckle	39	6.3
	Winter	Azure‐winged Magpie	43	3.2	Azure‐winged Magpie—Chinese ash	11	1.8

Abbreviation: Inc = incidence.

The species composition of birds was more similar in months belonging to the same season, both at the same site or across sites (Table S5). For example, the species composition of birds in October at BPAF was more similar to that in November at the same site (JI = 0.5) than that in other months. It was also more comparable to that in the same month at BAFLA (JI = 0.67) and THU (JI = 0.6) than those in the winter months. In some months, species other than the dominant species at the seasonal scale took up the top position (Table S6). For example, the Chinese Blackbird had the highest number of individuals in October (13, 1.0%) at BAFLA. At BPAF, the Brambling was the top bird species in January (20, 1.5%). In December, the Tree Sparrow took the top spot at THU (25, 1.9%).

The composition of bird–fruiting tree interaction pairs was also more similar in months belonging to the same season. In addition, the composition of bird–fruiting tree interaction pairs in months in the spring and summer, as well as in months in the autumn and winter, was more similar than those in months belonging to different groups (Table S7). At the monthly scale, some months were also dominated by bird‐fruiting tree pairs different from those at the seasonal scale (Table S8). For example, Brambling–Shantung Maple was the top interaction pair (11, 1.8%) in January at BAFLA. Brambling–Chinese Juniper led in December (13, 2.1%) at BPAF. At THU, the interaction between Tree Sparrow and Date‐plum was ranked the top (9, 1.5%) in December.

Bird species and their interactions with fruiting trees varied at different times of the day. In the autumn and winter, the two seasons that had the bulk of interactions, species other than Azur‐winged Magpie were more active in the early hours at BPAF and THU (Table S9). However, the Azur‐winged Magpie gradually took up the top positions in the late part of the day. The dominant bird‐fruit tree interaction pairs were similar to those at the seasonal scale. Some unique combinations were revealed with the finer time scale.

### 3.4 Temporal Variation in the Bird‐Tree Networks Across Sites

Annually, the interaction network at THU was characterized by the highest values of interaction diversity and interaction evenness among the three sites. In contrast, BPAF showed the lowest values of interaction diversity and interaction evenness, but the highest nestedness value (Table 5). The network structure varied at the seasonal scale. Especially, nestedness at BPAF exhibited marked temporal fluctuations; the value in the autumn was five times that in the winter. The network structure at BAFLA showed a moderate variation. The variation of network structure at THU was between the other two sites.

**TABLE 5:** Seasonal and annual values of four network metrics across the three sites.

Time	Location	Nestedness	Modularity	Interaction diversity	Interaction evenness
Annual	BAFLA	20.98	0.46	2.25	0.49
	BPAF	32.41	0.31	1.87	0.48
	THU	26.31	0.40	2.56	0.57
Spring	BAFLA	NA	NA	0.67	0.97
	BPAF	NA	NA	0.00	NA
	THU	NA	NA	0.00	NA
Summer	BAFLA	NA	NA	0.63	0.92
	BPAF	NA	NA	NA	NA
	THU	0.00	0.22	0.90	0.50
Autumn	BAFLA	23.52	0.37	1.56	0.45
	BPAF	61.11	0.10	0.86	0.35
	THU	16.67	0.28	1.55	0.54
Winter	BAFLA	19.78	0.47	2.63	0.62
	BPAF	15.22	0.06	1.95	0.65
	THU	20.00	0.41	2.10	0.59

## 4 Discussion

### 4.1 Temporal Patterns of Frugivory Events and Bird and Tree Species Involved

A distinctive seasonal pattern of avian frugivory was identified in this study. Most frugivory activities occurred in the autumn and winter, while the activities were low in the spring and summer. The pattern was consistent across the three sites. The same pattern has been observed previously in Beijing (He et al. 2021; Yin et al. 2023) and other cities located in the temperate climate zone (Caula et al. 2014; Suhonen et al. 2017). This seasonal pattern aligns with fruiting phenology peaking in late summer and autumn in cities within temperate climate zones. Additionally, migratory fueling increases frugivory during stopovers in these seasons. In contrast, during spring and summer, many urban birds shift to alternative foods (e.g., insects), so frugivory remains low. The observed pattern aligns with optimal foraging driven by seasonal energy constraints, with birds focusing on energy‐rich fruits in autumn and winter and decreasing frugivory in spring as alternative foods become more accessible. Additionally, competition may increase as fruits are exhausted in late winter (Hart et al. 2013; Pyke et al. 1977). However, generalist species like the Azure‐winged Magpie still contribute to avian frugivory in urban areas by consuming overwintered or unripe fruits. Additionally, the high frequency of frugivory events on certain days was driven by migratory bird species such as the Hawfinch and the Brambling, which together accounted for 26.9% of all frugivory events. Our findings corroborate the view that large‐scale temporal drivers such as fruiting phenology and migratory cycles still exert significant influence on frugivore activities in urban environments (Caula et al. 2014; Gallinat et al. 2020; He et al. 2021). Benefiting from the consistent monitoring, we also found that some species contributed to the frugivory events in the spring and summer, such as Azure‐winged Magpie, Oriental Magpie, and Spotted Dove. These species are considered generalists in the urban ecosystem (Alba et al. 2025; Lu et al. 2025). Abundant and generalist species such as the Azure‐winged Magpie and Hawfinch contributed most of the foraging events across the sites, while less common species like the Dusky Thrush were involved in a smaller percentage of interactions. The fruits they consumed include those left from the last autumn, like in Rockspray cotoneaster, and unripe fruits from species such as Atropurpurea flowering plum. The generalist birds were likely trying to get some key nutrients from fruits, such as vitamins, even when fruits are immature and other food resources (e.g., insects) are abundant in summer (Blendinger et al. 2015). Finally, the higher dissimilarity in the composition of interaction pairs than the species composition across the three sites indicated that the same bird species interacted with different tree species with different intensity. Therefore, focusing on the composition of bird species and fruiting tree species alone cannot paint a whole picture of frugivory.

Frugivory activities displayed more variation at the monthly scale at the three sites. A consistent pattern was the gradual decline in frugivory events in the three winter months. There are two possible reasons for this pattern. First, the number of fruits hanging on the branches decreased due to bird consumption and natural forces such as winds. Second, food availability increases as it gets closer to spring. For example, birds were found to eat tree sprouts and sap flows in early spring in Beijing (Yin et al. 2023). No frugivory events were recorded in August and September at the three sites. This period is the end of the breeding season and the molting time in Beijing; birds may prefer foods with high protein, such as insects (Zuckerberg et al. 2016). Also, most migratory birds have not reached Beijing yet.

Besides the two consistent patterns, considerable variation among sites was observed at the monthly scale. At BPAF, no frugivory events were observed during the summer months despite ongoing monitoring. This might be due to the relatively small number of fruiting tree species present at this site. Research on the Beijing plain forest project has shown that the tree plantations have a simple structure and are dominated by species such as Chinese white poplar (*Populus × tomentosa*) and Japanese pagoda tree ( *Styphnolobium japonicum* ) (Zhao et al. 2024). They are not considered a major fruit resource for frugivorous birds in Beijing. The concentrated pattern observed at this site contrasts with natural forests, where greater tree species diversity and varied fruiting times usually support longer periods of frugivory (Ramos‐Robles et al. 2018; Singh and Kushwaha 2006). Examining bird‐fruiting tree interactions at a monthly scale revealed major interaction pairs concealed at the seasonal level. For example, Brambling—Shantung Maple in January at BAFLA, Brambling—Chinese Juniper in December at BPAF, and Tree Sparrow—Date‐plum in December at THU were identified in this study. The monthly variation in frugivory activities and the involved bird and tree species across the three sites showed that local‐scale conditions modified the large‐scale temporal drivers.

The modification effect is more substantial on the diurnal rhythm. At BAFLA and BPAF, frugivory events spread out during the day, with peaks both in the morning and the afternoon. In contrast, frugivory events are concentrated in the morning at THU and remain low for most of the day. As a suburban forest plantation, human activities at BPAF were understandably low. While BAFLA is located in a dense urban area, its garden is at a distance from the main buildings. The small size of working professionals also contributes to predictable and low human activities in the garden. Besides, localized fruit aggregation likely enhanced detectability and foraging efficiency, as birds closely track fruit in clustered but predictable environments (Saracco et al. 2004). The situation was quite different at THU, where about 80,000 students and staff are using the campus all day. Therefore, for most of the time, the frugivory activities remained low. Urban birds have been observed to forage in sheltered microhabitats as a behavioral response to persistent disturbance (Yang et al. 2024). This may explain the low detectability of frugivory events at THU. These diurnal patterns were different from observations in forest ecosystems, where most frugivory occurs in the morning, as fruits become accessible and daylight increases, facilitating efficient foraging (Bhanda et al. 2025; Bonter et al. 2013; Graham et al. 2002). Seasonal shifts in daylight hours may also affect the observed daily patterns, as fewer daylight hours in winter could reduce foraging opportunities. The differences highlight the critical role of human activities in structuring frugivory dynamics in urban habitats.

### 4.2 Features of Bird‐Fruiting Tree Interaction Networks

Distinct structural characteristics in bird–fruiting tree interaction networks were identified across the three sites. Unlike natural forest ecosystems, which typically display high nestedness and moderate modularity due to stable and diverse mutualistic interactions (Bascompte et al. 2003; Schleuning et al. 2011), urban networks appear more variable and site‐specific. Such differences likely reflect the altered species composition, phenological mismatches, and fragmented habitats in urban systems (Li et al. 2022). BAFLA, characterized by aggregated fruiting patches and a high density of productive species such as *Malus* spp., showed relatively high modularity and interaction evenness. Modularity has been associated with greater ecological stability by confining perturbations within modules (Grilli et al. 2016; Robinson et al. 2020). THU had high interaction diversity and evenness, and moderate modularity, suggesting a compartmentalized and stable interaction pattern (Grilli et al. 2016). The pattern is likely supported by a diverse assemblage of tree species with prolonged fruiting phenology at the site. Although frugivory frequency was relatively low due to higher human activity, the evenly distributed interactions across species indicate that functional diversity was maintained through a rich and temporally extended fruit supply. In contrast, BPAF, with fewer fruiting tree species and simplified vegetation structure, showed the lowest modularity and the highest nestedness. This indicates a centralized core–periphery structure dominated by generalists and potentially more vulnerable to disturbance (Bascompte et al. 2003). These findings align with previous studies suggesting that network structure reflects biotic composition and spatial configuration of resources (Ferger et al. 2014; Li et al. 2022).

Interaction networks at all three sites exhibited seasonal patterns. This was evidenced by increased interaction diversity and evenness at all three sites, lowered nestedness at BAFLA and BPAF, and increased modularity values at BAFLA and THU between autumn and winter. Those changes together indicated a seasonal shift toward more complex and differentiated network organization. These patterns likely reflect the combined effects of increased reliance on fruits as food and the arrival of migratory frugivores, many of which reach Beijing between September and December. Similar seasonal enhancements in network structure have been observed in natural forests, where synchronized fruiting and migration pulses promote interaction richness and organization (Ramos‐Robles et al. 2018; Rutt and Stouffer 2021).

### 4.3 Implications and Limitations of the Current Study

Findings from this study are useful for guiding actions to preserve frugivorous birds and avian frugivory in urban environments. Fruiting tree species richness and abundance, and human disturbances, were found to affect the types and intensity of frugivory activities. Therefore, measures to add more fruiting trees and reduce human disturbances can be implemented to enhance avian frugivory. Moreover, our results clearly showed that bird‐fruiting tree interaction pairs varied in different seasons and months. It is important to match the fruiting time of trees to specific bird species, especially the migratory bird species. Therefore, having a consistent supply of fruits is key to satisfying the needs of different bird species. Urban forests serve as important feeding sites for birds, but birds can be better supported when fruit availability is stable and nutritionally sufficient (Maruyama et al. 2019; Peters et al. 2010). Finally, when evaluating the success of projects to enhance avian frugivory, it is important to pay attention to the types of birds and the intensity of interaction rather than focusing on numbers only. Our results show that a large number of generalist species were engaged in frugivory events. Therefore, using the number of birds feeding on fruit trees as an indicator can be misleading.

Our findings also have important implications for studies of avian frugivory in urban environments. Previous studies conducted in Beijing all relied on field surveys conducted at different time intervals, from once a month (Yin et al. 2023) to once a week (He et al. 2021). All were carried out in the morning before 11 a.m. Our results show that this conventional survey method is not able to capture the whole temporal dynamic of avian frugivory in urban environments. The high daily variation of frugivory events means that the survey result will be severely impacted by the selection of field days. Furthermore, more than half of frugivory events were recorded after 11 a.m. at our study sites. Restricting the survey to the morning helps to standardize the field protocol, but important frugivory events will be missed. For example, the phenomenon that generalists gradually dominate frugivory events in a day was discovered in this study. Moreover, camera trapping can capture both interactions and the number of birds involved in the interactions. The latter was challenging to count accurately (Norouzzadeh et al. 2018). Therefore, it is essential to combine the strength of traditional field surveys and arboreal camera trapping in future studies of avian frugivory in urban environments (Nordin et al. 2025). These findings also guide several management actions in urban forests. Bird‐friendly zones around key winter‐fruiting trees, with setback buffers from main paths and student activity areas, should be established whenever possible. If spaces cannot be allocated for bird‐friendly zones, using signage to warn people of disturbances at foraging hotspots can be an alternative. Additionally, vegetation management measures such as providing a continuous fruit supply across seasons using native and winter‐fruiting species, and maintaining shrub layers that screen human presence while maintaining sightlines for safety, can be implemented. Park maintenance and public events should be scheduled away from peak foraging times and high‐use fruit patches identified during monitoring.

While our study provides novel insights into the temporal dynamics of avian frugivory events in urban environments, several limitations should be considered when applying the findings from this study. This study covered three sites along an urban‐disturbance gradient. We focused on revealing the temporal patterns of avian frugivory in the urban environment, which was made possible by the intensive monitoring methods adopted in the current study. However, we are keenly aware that the small sample size and the limited spatial coverage restricted the representativeness and generalizability of the findings, especially regarding their spatial variation. We hope for collaboration with other researchers to expand the number and spatial distribution of sites across urban gradients to improve the representativeness and generalizability. Additionally, although cameras were placed at least 2–3 m apart to minimize overlapping fields of view, some possibility of double‐counting cannot be completely excluded. Camera traps monitored only a subset of fruiting trees at each site, so interactions might have been missed because of the limited field of view and foliage occlusion. Although infrared cameras provided high resolution foraging data, they may have failed to detect frugivory events occurring under dense foliage or outside the field of view, particularly in structurally complex habitats (Zhu et al. 2022). This limitation could be addressed by incorporating direct field observations or acoustic sensors to enhance detection reliability and behavioral classification accuracy (Yang et al. 2024).

## 5 Conclusion

Understanding the pattern of avian frugivory and its influencing factors is essential for maintaining frugivorous bird diversity in urban environments. Our study demonstrates that large‐scale fruiting phenology and bird migratory events impact frugivorous bird richness and the number of frugivory events. These effects were modified at finer spatial–temporal scales by variation in human disturbance and fruit tree species richness. The influences of these large‐ and small‐scale factors were also evident in the structure of the network of birds and fruiting trees. Our findings highlight the importance of examining avian frugivory at high temporal resolution in urban environments. Future studies should combine the strengths of arboreal camera trapping and field surveys to gain a deeper understanding of avian frugivory in urban forests.

## Author Contributions

**Xinyi Liu:** conceptualization (equal), data curation (equal), formal analysis (equal), investigation (equal), methodology (equal), visualization (equal), writing – original draft (lead). **Xudong Yang:** data curation (equal), investigation (equal). **Jing Zhou:** data curation (equal), investigation (equal). **Xinyu Li:** data curation (equal), investigation (equal). **Zhitao Guo:** data curation (equal), investigation (equal). **Jun Yang:** conceptualization (lead), funding acquisition (lead), methodology (equal), project administration (lead), supervision (lead), writing – original draft (equal).

## Funding

This study was supported by a grant from the National Natural Science Foundation of China (Grant No. 32171542).

## Conflicts of Interest

The authors declare no conflicts of interest.

## Supporting information


**Appendix S1:** ece372699‐sup‐0001‐AppendixS1.docx.

## Data Availability

The datasets and code have been archived in figshare: https://doi.org/10.6084/m9.figshare.29940176.
