# Supplementary material for: Patterns of Avian Frugivory in Beijing's Urban Forest Across Multiple Temporal Scales
Source: Ecol Evol. 2025 Dec 17;15(12):e72699. doi: 10.1002/ece3.72699 (PMC12710094; doi:10.1002/ece3.72699)
Supplement: Supplementary file 1 — Appendix S1: ece372699‐sup‐0001‐AppendixS1.docx. [file ECE3-15-e72699-s001.docx]

Supplementary file for *Patterns of avian frugivory in Beijing’s urban forest across multiple temporal scales*

Table S1. Taxonomic information of recorded fruiting tree species and frugivorous bird species.

| Type | Scientific name | Common name | Origin | Fruiting time | Time in Beijing |
| --- | --- | --- | --- | --- | --- |
| Tree | *Lonicera maackii* | Amur honeysuckle | Natural distribution | Sep-Oct | - |
| Tree | *Euonymus laxiflorus* | Loose-flowered Euonymus | Native in China | Aug-Oct | - |
| Tree | *Juniperus chinensis* | Chinese juniper | Natural distribution | Jan-Dec | - |
| Tree | *Platycladus orientalis* | Oriental arborvitae | Natural distribution | Jan-Dec | - |
| Tree | *Diospyros lotus* | Date-plum | Natural distribution | Oct-Nov | - |
| Tree | *Fraxinus chinensis* | Chinese ash | Native in China | Sep-Oct | - |
| Tree | *Pinus tabuliformis* | Manchurian red pine | Natural distribution | Jan-Dec | - |
| Tree | *Cotoneaster horizontalis* | Rockspray cotoneaster | Native in China | Sep-Mar (next year) | - |
| Tree | *Malus* spp. | Crabapple | Cultivars | Jun-Mar (next year) | - |
| Tree | *Prunus cerasifera* 'Atropurpurea' | Atropurpurea flowering plum | Native in China | Apr-Aug | - |
| Tree | *Acer negundo* | Manitoba maple | Exotic | Sep | - |
| Tree | *Acer truncatum* | Shantung maple | Natural distribution | Aug-Sep | - |
| Bird | *Spilopelia chinensis* | Spotted Dove | Resident | - | Jan-Dec |
| Bird | *Streptopelia orientalis* | Oriental Turtle Dove | Resident | - | Jan-Dec |
| Bird | *Cyanopica cyanus* | Azure-winged Magpie | Resident | - | Jan-Dec |
| Bird | *Pica serica* | Oriental Magpie | Resident | - | Jan-Dec |
| Bird | *Urocissa erythroryncha* | Red-billed Blue Magpie | Resident | - | Jan-Dec |
| Bird | *Emberiza elegans* | Yellow-throated Bunting | Migratory | - | Mar-Jun, Aug-Nov |
| Bird | *Coccothraustes coccothraustes* | Hawfinch | Migratory | - | Jan-Jun, Oct-Dec |
| Bird | *Eophona migratoria* | Chinese Grosbeak | Migratory | - | Jan-May, Aug-Dec |
| Bird | *Fringilla montifringilla* | Brambling | Migratory | - | Jan-May, Oct-Dec |
| Bird | *Parus minor* | Japanese Tit | Resident | - | Jan-Dec |
| Bird | *Poecile palustris* | Marsh Tit | Resident | - | Jan-Dec |
| Bird | *Passer montanus* | Tree Sparrow | Resident | - | Jan-Dec |
| Bird | *Dendrocopos major* | Great Spotted Woodpecker | Resident | - | Jan-Dec |
| Bird | *Pycnonotus sinensis* | Light-vented Bulbul | Resident | - | Jan-Dec |
| Bird | *Spodiopsar cineraceus* | White-cheeked Starling | Resident | - | Jan-Dec |
| Bird | *Turdus mandarinus* | Chinese Blackbird | Resident | - | Jan-Dec |
| Bird | *Turdus naumanni* | Naumann's Thrush | Migratory | - | Jan-Jun, Sep-Dec |
| Bird | *Turdus ruficollis* | Red-throated Thrush | Migratory | - | Jan-Mar, Nov-Dec |
| Bird | *Turdus eunomus* | Dusky Thrush | Migratory | - | Jan-Jun, Sep-Dec |

Table S2 Analysis of variance table for frugivory events in different months and sites

|  | Df | R Sum Sq | R Mean Sq | Iter | Pr |
| --- | --- | --- | --- | --- | --- |
| Location | 2 | 888.7 | 444.33 | 5000 | 0.0054** |
| Month | 8 | 16432.4 | 2054..04 | 5000 | 0.0056** |
| Location:Month | 11 | 9872.7 | 897.51.5 | 5000 | 0.0100** |
| Residuals | 0 | 0.0 | NaN |  |  |

Significance code: 0 ‘***’; 0.001 ‘**’; 0.01 ‘*’; <0.05 ‘.’

Table S3 Analysis of variance table for frugivory events in different dates and sites

|  | Df | R Sum Sq | R Mean Sq | Iter | Pr |
| --- | --- | --- | --- | --- | --- |
| Location | 2 | 580.7 | 290.333 | 5000 | <2.2e^-16^*** |
| Date | 129 | 3462.2 | 28.234 | 5000 | <2.2e^-16^*** |
| Location:Date | 48 | 1687.1 | 35.147 | 5000 | <2.2e^-16^*** |
| Residuals | 0 | 0.0 | NaN |  |  |

Significance code: 0 ‘***’; 0.001 ‘**’; 0.01 ‘*’; <0.05 ‘.’

Table S4 Analysis of variance table for frugivory events in different hours, seasons, and sites

|  | Df | R Sum Sq | R Mean Sq | Iter | Pr |
| --- | --- | --- | --- | --- | --- |
| Location | 2 | 35.89 | 17.943 | 5000 | 0.0098** |
| Season | 3 | 29.68 | 9.892 | 5000 | 0.0112* |
| Location:Season | 5 | 96.17 | 19.233 | 5000 | 0.0112* |
| Hour | 14 | 644.71 | 46.051 | 5000 | 0.0112* |
| Location:Hour | 20 | 739.06 | 36.953 | 5000 | 0.0088** |
| Season:Hour | 19 | 315.39 | 16.599 | 5000 | 0.0112* |
| Location:Season:Hour | 17 | 553.81 | 32.577 | 5000 | 0.0086** |
| Residuals | 0 | 0.0 | NaN |  |  |

Significance code: 0 ‘***’; 0.001 ‘**’; 0.01 ‘*’; <0.05 ‘.’

Table S5 The dissimilarity of bird species composition at the three sites in different months measured in Jaccard dissimilarity coefficients (0= identical sets; 1= no similarity)

| Month/Site | 10_BPAF | 11_BPAF | 4_BPAF | 12_BAFLA | 11_BAFLA | 10_BAFLA | 4_BAFLA | 6_BAFLA | 12_BPAF | 1_BPAF | 2_BPAF | 2_THU | 3_THU | 12_THU | 1_THU | 10_THU | 6_THU | 7_THU | 11_THU | 1_BAFLA | 7_BAFLA | 2_BAFLA |
| --- | --- | --- | --- | --- | --- | --- | --- | --- | --- | --- | --- | --- | --- | --- | --- | --- | --- | --- | --- | --- | --- | --- |
| 10_BPAF | 0.00 | 0.50 | 1.00 | 0.83 | 0.75 | 0.67 | 0.50 | 0.50 | 0.86 | 0.71 | 0.67 | 0.50 | 0.50 | 0.50 | 0.80 | 0.60 | 1.00 | 0.67 | 0.60 | 1.00 | 0.50 | 1.00 |
| 11_BPAF | 0.50 | 0.00 | 0.75 | 0.77 | 0.80 | 0.80 | 0.75 | 0.75 | 0.89 | 0.78 | 0.75 | 0.75 | 0.75 | 0.67 | 0.86 | 0.71 | 1.00 | 0.80 | 0.71 | 1.00 | 0.75 | 1.00 |
| 4_BPAF | 1.00 | 0.75 | 0.00 | 0.92 | 1.00 | 1.00 | 1.00 | 1.00 | 1.00 | 1.00 | 1.00 | 1.00 | 1.00 | 1.00 | 1.00 | 1.00 | 1.00 | 1.00 | 1.00 | 1.00 | 1.00 | 1.00 |
| 12_BAFLA | 0.83 | 0.77 | 0.92 | 0.00 | 0.46 | 0.83 | 0.92 | 0.92 | 0.62 | 0.64 | 0.62 | 0.92 | 0.92 | 0.67 | 0.67 | 0.69 | 0.83 | 0.83 | 0.79 | 0.83 | 0.92 | 1.00 |
| 11_BAFLA | 0.75 | 0.80 | 1.00 | 0.46 | 0.00 | 0.75 | 0.88 | 0.88 | 0.73 | 0.75 | 0.73 | 0.88 | 0.88 | 0.80 | 0.80 | 0.70 | 0.89 | 0.89 | 0.56 | 0.75 | 0.88 | 0.89 |
| 10_BAFLA | 0.67 | 0.80 | 1.00 | 0.83 | 0.75 | 0.00 | 0.50 | 0.50 | 0.86 | 0.88 | 0.86 | 0.50 | 0.50 | 0.80 | 0.80 | 0.60 | 1.00 | 0.67 | 0.60 | 1.00 | 0.50 | 1.00 |
| 4_BAFLA | 0.50 | 0.75 | 1.00 | 0.92 | 0.88 | 0.50 | 0.00 | 0.00 | 0.83 | 0.86 | 0.83 | 0.00 | 0.00 | 0.75 | 0.75 | 0.80 | 1.00 | 0.50 | 0.80 | 1.00 | 0.00 | 1.00 |
| 6_BAFLA | 0.50 | 0.75 | 1.00 | 0.92 | 0.88 | 0.50 | 0.00 | 0.00 | 0.83 | 0.86 | 0.83 | 0.00 | 0.00 | 0.75 | 0.75 | 0.80 | 1.00 | 0.50 | 0.80 | 1.00 | 0.00 | 1.00 |
| 12_BPAF | 0.86 | 0.89 | 1.00 | 0.62 | 0.73 | 0.86 | 0.83 | 0.83 | 0.00 | 0.70 | 0.67 | 0.83 | 0.83 | 0.75 | 0.57 | 0.90 | 0.86 | 0.67 | 0.90 | 0.67 | 0.83 | 1.00 |
| 1_BPAF | 0.71 | 0.78 | 1.00 | 0.64 | 0.75 | 0.88 | 0.86 | 0.86 | 0.70 | 0.00 | 0.14 | 0.86 | 0.86 | 0.63 | 0.78 | 0.80 | 1.00 | 0.88 | 0.80 | 0.88 | 0.86 | 1.00 |
| 2_BPAF | 0.67 | 0.75 | 1.00 | 0.62 | 0.73 | 0.86 | 0.83 | 0.83 | 0.67 | 0.14 | 0.00 | 0.83 | 0.83 | 0.57 | 0.75 | 0.78 | 1.00 | 0.86 | 0.78 | 0.86 | 0.83 | 1.00 |
| 2_THU | 0.50 | 0.75 | 1.00 | 0.92 | 0.88 | 0.50 | 0.00 | 0.00 | 0.83 | 0.86 | 0.83 | 0.00 | 0.00 | 0.75 | 0.75 | 0.80 | 1.00 | 0.50 | 0.80 | 1.00 | 0.00 | 1.00 |
| 3_THU | 0.50 | 0.75 | 1.00 | 0.92 | 0.88 | 0.50 | 0.00 | 0.00 | 0.83 | 0.86 | 0.83 | 0.00 | 0.00 | 0.75 | 0.75 | 0.80 | 1.00 | 0.50 | 0.80 | 1.00 | 0.00 | 1.00 |
| 12_THU | 0.50 | 0.67 | 1.00 | 0.67 | 0.80 | 0.80 | 0.75 | 0.75 | 0.75 | 0.63 | 0.57 | 0.75 | 0.75 | 0.00 | 0.67 | 0.71 | 0.80 | 0.50 | 0.71 | 1.00 | 0.75 | 1.00 |
| 1_THU | 0.80 | 0.86 | 1.00 | 0.67 | 0.80 | 0.80 | 0.75 | 0.75 | 0.57 | 0.78 | 0.75 | 0.75 | 0.75 | 0.67 | 0.00 | 0.88 | 0.80 | 0.50 | 0.88 | 1.00 | 0.75 | 1.00 |
| 10_THU | 0.60 | 0.71 | 1.00 | 0.69 | 0.70 | 0.60 | 0.80 | 0.80 | 0.90 | 0.80 | 0.78 | 0.80 | 0.80 | 0.71 | 0.88 | 0.00 | 1.00 | 0.83 | 0.33 | 1.00 | 0.80 | 1.00 |
| 6_THU | 1.00 | 1.00 | 1.00 | 0.83 | 0.89 | 1.00 | 1.00 | 1.00 | 0.86 | 1.00 | 1.00 | 1.00 | 1.00 | 0.80 | 0.80 | 1.00 | 0.00 | 0.67 | 1.00 | 1.00 | 1.00 | 1.00 |
| 7_THU | 0.67 | 0.80 | 1.00 | 0.83 | 0.89 | 0.67 | 0.50 | 0.50 | 0.67 | 0.88 | 0.86 | 0.50 | 0.50 | 0.50 | 0.50 | 0.83 | 0.67 | 0.00 | 0.83 | 1.00 | 0.50 | 1.00 |
| 11_THU | 0.60 | 0.71 | 1.00 | 0.79 | 0.56 | 0.60 | 0.80 | 0.80 | 0.90 | 0.80 | 0.78 | 0.80 | 0.80 | 0.71 | 0.88 | 0.33 | 1.00 | 0.83 | 0.00 | 1.00 | 0.80 | 0.83 |
| 1_BAFLA | 1.00 | 1.00 | 1.00 | 0.83 | 0.75 | 1.00 | 1.00 | 1.00 | 0.67 | 0.88 | 0.86 | 1.00 | 1.00 | 1.00 | 1.00 | 1.00 | 1.00 | 1.00 | 1.00 | 0.00 | 1.00 | 1.00 |
| 7_BAFLA | 0.50 | 0.75 | 1.00 | 0.92 | 0.88 | 0.50 | 0.00 | 0.00 | 0.83 | 0.86 | 0.83 | 0.00 | 0.00 | 0.75 | 0.75 | 0.80 | 1.00 | 0.50 | 0.80 | 1.00 | 0.00 | 1.00 |
| 2_BAFLA | 1.00 | 1.00 | 1.00 | 1.00 | 0.89 | 1.00 | 1.00 | 1.00 | 1.00 | 1.00 | 1.00 | 1.00 | 1.00 | 1.00 | 1.00 | 1.00 | 1.00 | 1.00 | 0.83 | 1.00 | 1.00 | 0.00 |

Table S6 Bird species and their abundance in each month at the three sites

| Location | Month | Bird species | Abundance | Percentage |
| --- | --- | --- | --- | --- |
| BAFLA | 1 | Brambling | 54 | 4.00 |
| BAFLA | 1 | Hawfinch | 4 | 0.30 |
| BAFLA | 2 | Marsh Tit | 3 | 0.22 |
| BAFLA | 2 | Great Spotted Woodpecker | 1 | 0.07 |
| BAFLA | 4 | Azure-Winged Magpie | 5 | 0.37 |
| BAFLA | 6 | Azure-Winged Magpie | 3 | 0.22 |
| BAFLA | 7 | Azure-Winged Magpie | 4 | 0.30 |
| BAFLA | 10 | Chinese Blackbird | 13 | 0.96 |
| BAFLA | 10 | Azure-Winged Magpie | 5 | 0.37 |
| BAFLA | 11 | Hawfinch | 210 | 15.57 |
| BAFLA | 11 | Brambling | 136 | 10.08 |
| BAFLA | 11 | Azure-Winged Magpie | 14 | 1.04 |
| BAFLA | 11 | Chinese Blackbird | 3 | 0.22 |
| BAFLA | 11 | Spotted Dove | 3 | 0.22 |
| BAFLA | 11 | Light-Vented Bulbul | 2 | 0.15 |
| BAFLA | 11 | Marsh Tit | 1 | 0.07 |
| BAFLA | 11 | Naumann's Thrush | 1 | 0.07 |
| BAFLA | 12 | Azure-Winged Magpie | 21 | 1.56 |
| BAFLA | 12 | Brambling | 15 | 1.11 |
| BAFLA | 12 | Hawfinch | 13 | 0.96 |
| BAFLA | 12 | Naumann's Thrush | 6 | 0.44 |
| BAFLA | 12 | Red-Throated Thrush | 5 | 0.37 |
| BAFLA | 12 | Spotted Dove | 5 | 0.37 |
| BAFLA | 12 | Light-Vented Bulbul | 4 | 0.30 |
| BAFLA | 12 | White Cheeked Starling | 4 | 0.30 |
| BAFLA | 12 | Red-Billed Blue Magpie | 2 | 0.15 |
| BAFLA | 12 | Chinese Blackbird | 1 | 0.07 |
| BAFLA | 12 | Oriental Magpie | 1 | 0.07 |
| BAFLA | 12 | Tree Sparrow | 1 | 0.07 |
| BPAF | 1 | Brambling | 20 | 1.48 |
| BPAF | 1 | Tree Sparrow | 12 | 0.89 |
| BPAF | 1 | Light-Vented Bulbul | 9 | 0.67 |
| BPAF | 1 | Azure-Winged Magpie | 6 | 0.44 |
| BPAF | 1 | Japanese Tit | 5 | 0.37 |
| BPAF | 1 | Red-Throated Thrush | 1 | 0.07 |
| BPAF | 1 | Yellow-Throated Bunting | 1 | 0.07 |
| BPAF | 2 | Light-Vented Bulbul | 25 | 1.85 |
| BPAF | 2 | Azure-Winged Magpie | 2 | 0.15 |
| BPAF | 2 | Japanese Tit | 2 | 0.15 |
| BPAF | 2 | Brambling | 1 | 0.07 |
| BPAF | 2 | Red-Throated Thrush | 1 | 0.07 |
| BPAF | 2 | Tree Sparrow | 1 | 0.07 |
| BPAF | 4 | Red-Billed Blue Magpie | 1 | 0.07 |
| BPAF | 10 | Azure-Winged Magpie | 45 | 3.34 |
| BPAF | 10 | Light-Vented Bulbul | 4 | 0.30 |
| BPAF | 11 | Azure-Winged Magpie | 359 | 26.61 |
| BPAF | 11 | Light-Vented Bulbul | 9 | 0.67 |
| BPAF | 11 | Dusky Thrush | 5 | 0.37 |
| BPAF | 11 | Red-Billed Blue Magpie | 1 | 0.07 |
| BPAF | 12 | Azure-Winged Magpie | 67 | 4.97 |
| BPAF | 12 | Brambling | 36 | 2.67 |
| BPAF | 12 | Red-Throated Thrush | 11 | 0.82 |
| BPAF | 12 | Hawfinch | 6 | 0.44 |
| BPAF | 12 | Oriental Magpie | 1 | 0.07 |
| BPAF | 12 | Oriental Turtle Dove | 1 | 0.07 |
| THU | 1 | Azure-Winged Magpie | 12 | 0.89 |
| THU | 1 | Naumann's Thrush | 1 | 0.07 |
| THU | 1 | Oriental Magpie | 1 | 0.07 |
| THU | 1 | Red-Throated Thrush | 1 | 0.07 |
| THU | 2 | Azure-Winged Magpie | 9 | 0.67 |
| THU | 3 | Azure-Winged Magpie | 3 | 0.22 |
| THU | 6 | Oriental Magpie | 4 | 0.30 |
| THU | 6 | Spotted Dove | 2 | 0.15 |
| THU | 7 | Azure-Winged Magpie | 2 | 0.15 |
| THU | 7 | Oriental Magpie | 1 | 0.07 |
| THU | 10 | Light-Vented Bulbul | 41 | 3.04 |
| THU | 10 | Azure-Winged Magpie | 14 | 1.04 |
| THU | 10 | Chinese Blackbird | 3 | 0.22 |
| THU | 10 | Chinese Grosbeak | 1 | 0.07 |
| THU | 10 | White Cheeked Starling | 1 | 0.07 |
| THU | 11 | Azure-Winged Magpie | 10 | 0.74 |
| THU | 11 | Light-Vented Bulbul | 5 | 0.37 |
| THU | 11 | Chinese Blackbird | 1 | 0.07 |
| THU | 11 | Chinese Grosbeak | 1 | 0.07 |
| THU | 11 | Marsh Tit | 1 | 0.07 |
| THU | 12 | Tree Sparrow | 25 | 1.85 |
| THU | 12 | Azure-Winged Magpie | 22 | 1.63 |
| THU | 12 | Oriental Magpie | 9 | 0.67 |
| THU | 12 | Light-Vented Bulbul | 7 | 0.52 |

Table S7 The dissimilarity of bird–fruiting tree interaction pairs at the three sites in different months measured in Bray-Curtis dissimilarity index (0= identical sets; 1= no similarity).

| Month/Site | 10_BPAF | 10_THU | 11_BPAF | 11_THU | 4_BAFLA | 6_BAFLA | 1_THU | 2_THU | 12_THU | 1_BPAF | 2_BPAF | 12_BPAF | 7_BAFLA | 7_THU | 10_BAFLA | 11_BAFLA | 12_BAFLA | 3_THU | 1_BAFLA | 2_BAFLA | 6_THU | 4_BPAF |
| --- | --- | --- | --- | --- | --- | --- | --- | --- | --- | --- | --- | --- | --- | --- | --- | --- | --- | --- | --- | --- | --- | --- |
| 10_BPAF | 0.00 | 0.70 | 0.65 | 0.67 | 0.88 | 0.94 | 1.00 | 1.00 | 1.00 | 1.00 | 1.00 | 1.00 | 0.81 | 0.94 | 0.78 | 0.84 | 0.58 | 1.00 | 1.00 | 1.00 | 1.00 | 1.00 |
| 10_THU | 0.70 | 0.00 | 0.79 | 0.75 | 0.94 | 0.97 | 1.00 | 0.97 | 1.00 | 1.00 | 1.00 | 1.00 | 0.90 | 0.97 | 0.84 | 0.90 | 0.82 | 0.97 | 1.00 | 1.00 | 1.00 | 1.00 |
| 11_BPAF | 0.65 | 0.79 | 0.00 | 0.91 | 0.97 | 0.99 | 1.00 | 1.00 | 1.00 | 1.00 | 1.00 | 1.00 | 0.96 | 0.99 | 0.94 | 0.90 | 0.81 | 1.00 | 1.00 | 1.00 | 1.00 | 1.00 |
| 11_THU | 0.67 | 0.75 | 0.91 | 0.00 | 0.79 | 0.88 | 1.00 | 1.00 | 1.00 | 1.00 | 1.00 | 1.00 | 0.65 | 0.88 | 0.74 | 0.92 | 0.82 | 1.00 | 1.00 | 1.00 | 1.00 | 1.00 |
| 4_BAFLA | 0.88 | 0.94 | 0.97 | 0.79 | 0.00 | 0.25 | 1.00 | 1.00 | 1.00 | 1.00 | 1.00 | 1.00 | 0.50 | 0.71 | 0.82 | 0.97 | 0.94 | 1.00 | 1.00 | 1.00 | 1.00 | 1.00 |
| 6_BAFLA | 0.94 | 0.97 | 0.99 | 0.88 | 0.25 | 0.00 | 1.00 | 1.00 | 1.00 | 1.00 | 1.00 | 1.00 | 0.67 | 0.60 | 0.90 | 0.98 | 0.97 | 1.00 | 1.00 | 1.00 | 1.00 | 1.00 |
| 1_THU | 1.00 | 1.00 | 1.00 | 1.00 | 1.00 | 1.00 | 0.00 | 0.79 | 0.84 | 1.00 | 1.00 | 1.00 | 1.00 | 1.00 | 1.00 | 1.00 | 1.00 | 1.00 | 1.00 | 1.00 | 1.00 | 1.00 |
| 2_THU | 1.00 | 0.97 | 1.00 | 1.00 | 1.00 | 1.00 | 0.79 | 0.00 | 0.95 | 1.00 | 1.00 | 1.00 | 1.00 | 1.00 | 1.00 | 1.00 | 0.97 | 0.83 | 1.00 | 1.00 | 1.00 | 1.00 |
| 12_THU | 1.00 | 1.00 | 1.00 | 1.00 | 1.00 | 1.00 | 0.84 | 0.95 | 0.00 | 1.00 | 1.00 | 1.00 | 1.00 | 1.00 | 1.00 | 1.00 | 1.00 | 1.00 | 1.00 | 1.00 | 1.00 | 1.00 |
| 1_BPAF | 1.00 | 1.00 | 1.00 | 1.00 | 1.00 | 1.00 | 1.00 | 1.00 | 1.00 | 0.00 | 0.45 | 0.63 | 1.00 | 1.00 | 1.00 | 1.00 | 1.00 | 1.00 | 1.00 | 1.00 | 1.00 | 1.00 |
| 2_BPAF | 1.00 | 1.00 | 1.00 | 1.00 | 1.00 | 1.00 | 1.00 | 1.00 | 1.00 | 0.45 | 0.00 | 0.86 | 1.00 | 1.00 | 1.00 | 1.00 | 1.00 | 1.00 | 1.00 | 1.00 | 1.00 | 1.00 |
| 12_BPAF | 1.00 | 1.00 | 1.00 | 1.00 | 1.00 | 1.00 | 1.00 | 1.00 | 1.00 | 0.63 | 0.86 | 0.00 | 1.00 | 1.00 | 1.00 | 1.00 | 1.00 | 1.00 | 1.00 | 1.00 | 1.00 | 1.00 |
| 7_BAFLA | 0.81 | 0.90 | 0.96 | 0.65 | 0.50 | 0.67 | 1.00 | 1.00 | 1.00 | 1.00 | 1.00 | 1.00 | 0.00 | 0.60 | 0.70 | 0.95 | 0.91 | 1.00 | 1.00 | 1.00 | 1.00 | 1.00 |
| 7_THU | 0.94 | 0.97 | 0.99 | 0.88 | 0.71 | 0.60 | 1.00 | 1.00 | 1.00 | 1.00 | 1.00 | 1.00 | 0.60 | 0.00 | 0.89 | 0.98 | 0.97 | 1.00 | 1.00 | 1.00 | 0.75 | 1.00 |
| 10_BAFLA | 0.78 | 0.84 | 0.94 | 0.74 | 0.82 | 0.90 | 1.00 | 1.00 | 1.00 | 1.00 | 1.00 | 1.00 | 0.70 | 0.89 | 0.00 | 0.89 | 0.85 | 1.00 | 1.00 | 1.00 | 1.00 | 1.00 |
| 11_BAFLA | 0.84 | 0.90 | 0.90 | 0.92 | 0.97 | 0.98 | 1.00 | 1.00 | 1.00 | 1.00 | 1.00 | 1.00 | 0.95 | 0.98 | 0.89 | 0.00 | 0.77 | 1.00 | 0.79 | 0.98 | 1.00 | 1.00 |
| 12_BAFLA | 0.58 | 0.82 | 0.81 | 0.82 | 0.94 | 0.97 | 1.00 | 0.97 | 1.00 | 1.00 | 1.00 | 1.00 | 0.91 | 0.97 | 0.85 | 0.77 | 0.00 | 0.97 | 1.00 | 1.00 | 1.00 | 1.00 |
| 3_THU | 1.00 | 0.97 | 1.00 | 1.00 | 1.00 | 1.00 | 1.00 | 0.83 | 1.00 | 1.00 | 1.00 | 1.00 | 1.00 | 1.00 | 1.00 | 1.00 | 0.97 | 0.00 | 1.00 | 1.00 | 1.00 | 1.00 |
| 1_BAFLA | 1.00 | 1.00 | 1.00 | 1.00 | 1.00 | 1.00 | 1.00 | 1.00 | 1.00 | 1.00 | 1.00 | 1.00 | 1.00 | 1.00 | 1.00 | 0.79 | 1.00 | 1.00 | 0.00 | 1.00 | 1.00 | 1.00 |
| 2_BAFLA | 1.00 | 1.00 | 1.00 | 1.00 | 1.00 | 1.00 | 1.00 | 1.00 | 1.00 | 1.00 | 1.00 | 1.00 | 1.00 | 1.00 | 1.00 | 0.98 | 1.00 | 1.00 | 1.00 | 0.00 | 1.00 | 1.00 |
| 6_THU | 1.00 | 1.00 | 1.00 | 1.00 | 1.00 | 1.00 | 1.00 | 1.00 | 1.00 | 1.00 | 1.00 | 1.00 | 1.00 | 0.75 | 1.00 | 1.00 | 1.00 | 1.00 | 1.00 | 1.00 | 0.00 | 1.00 |
| 4_BPAF | 1.00 | 1.00 | 1.00 | 1.00 | 1.00 | 1.00 | 1.00 | 1.00 | 1.00 | 1.00 | 1.00 | 1.00 | 1.00 | 1.00 | 1.00 | 1.00 | 1.00 | 1.00 | 1.00 | 1.00 | 1.00 | 0.00 |

Table S8 Bird and fruiting tree species interaction pairs and their frequencies in each month

| Location | Month | Bird-tree pair | Frequency | Percentage |
| --- | --- | --- | --- | --- |
| BAFLA | 1 | Brambling-Shantung maple | 11 | 1.78 |
| BAFLA | 1 | Hawfinch-Shantung maple | 4 | 0.65 |
| BAFLA | 2 | Marsh Tit-Manchurian red pine | 3 | 0.49 |
| BAFLA | 2 | Great Spotted Woodpecker-Manchurian red pine | 1 | 0.16 |
| BAFLA | 4 | Azure-Winged Magpie-Atropurpurea flowering plum | 3 | 0.49 |
| BAFLA | 4 | Azure-Winged Magpie-Malus spp. | 2 | 0.32 |
| BAFLA | 6 | Azure-Winged Magpie-Atropurpurea flowering plum | 2 | 0.32 |
| BAFLA | 6 | Azure-Winged Magpie-Malus spp. | 1 | 0.16 |
| BAFLA | 7 | Azure-Winged Magpie-Malus spp. | 3 | 0.49 |
| BAFLA | 10 | Chinese Blackbird-Malus spp. | 11 | 1.78 |
| BAFLA | 10 | Azure-Winged Magpie-Malus spp. | 5 | 0.81 |
| BAFLA | 10 | Chinese Blackbird-Amur honeysuckle | 1 | 0.16 |
| BAFLA | 11 | Hawfinch-Shantung maple | 62 | 10.03 |
| BAFLA | 11 | Brambling-Shantung maple | 40 | 6.47 |
| BAFLA | 11 | Azure-Winged Magpie-Malus spp. | 13 | 2.10 |
| BAFLA | 11 | Brambling-Malus spp. | 3 | 0.49 |
| BAFLA | 11 | Chinese Blackbird-Malus spp. | 3 | 0.49 |
| BAFLA | 11 | Hawfinch-Malus spp. | 2 | 0.32 |
| BAFLA | 11 | Light-Vented Bulbul-Malus spp. | 2 | 0.32 |
| BAFLA | 11 | Spotted Dove-Malus spp. | 2 | 0.32 |
| BAFLA | 11 | Marsh Tit-Manchurian red pine | 1 | 0.16 |
| BAFLA | 11 | Naumann's Thrush-Malus spp. | 1 | 0.16 |
| BAFLA | 12 | Azure-Winged Magpie-Malus spp. | 19 | 3.07 |
| BAFLA | 12 | Brambling-Malus spp. | 7 | 1.13 |
| BAFLA | 12 | Hawfinch-Rockspray cotoneaster | 5 | 0.81 |
| BAFLA | 12 | Naumann's Thrush-Malus spp. | 5 | 0.81 |
| BAFLA | 12 | Hawfinch-Malus spp. | 4 | 0.65 |
| BAFLA | 12 | Red-Throated Thrush-Rockspray cotoneaster | 4 | 0.65 |
| BAFLA | 12 | Hawfinch-Chinese ash | 3 | 0.49 |
| BAFLA | 12 | Light-Vented Bulbul-Malus spp. | 3 | 0.49 |
| BAFLA | 12 | Red-Billed Blue Magpie-Rockspray cotoneaster | 2 | 0.32 |
| BAFLA | 12 | Spotted Dove-Rockspray cotoneaster | 2 | 0.32 |
| BAFLA | 12 | White Cheeked Starling-Rockspray cotoneaster | 2 | 0.32 |
| BAFLA | 12 | Azure-Winged Magpie-Rockspray cotoneaster | 1 | 0.16 |
| BAFLA | 12 | Brambling-Rockspray cotoneaster | 1 | 0.16 |
| BAFLA | 12 | Chinese Blackbird-Malus spp. | 1 | 0.16 |
| BAFLA | 12 | Oriental Magpie-Malus spp. | 1 | 0.16 |
| BAFLA | 12 | Spotted Dove-Chinese ash | 1 | 0.16 |
| BAFLA | 12 | Tree Sparrow-Rockspray cotoneaster | 1 | 0.16 |
| BPAF | 1 | Azure-Winged Magpie-Chinese juniper | 6 | 0.97 |
| BPAF | 1 | Light-Vented Bulbul-Chinese juniper | 6 | 0.97 |
| BPAF | 1 | Brambling-Chinese juniper | 4 | 0.65 |
| BPAF | 1 | Japanese Tit-Chinese juniper | 4 | 0.65 |
| BPAF | 1 | Japanese Tit-Oriental arborvitae | 1 | 0.16 |
| BPAF | 1 | Red-Throated Thrush-Chinese juniper | 1 | 0.16 |
| BPAF | 1 | Tree Sparrow-Chinese juniper | 1 | 0.16 |
| BPAF | 1 | Tree Sparrow-Oriental arborvitae | 1 | 0.16 |
| BPAF | 1 | Yellow-Throated Bunting-Chinese juniper | 1 | 0.16 |
| BPAF | 2 | Light-Vented Bulbul-Chinese juniper | 15 | 2.43 |
| BPAF | 2 | Azure-Winged Magpie-Chinese juniper | 2 | 0.32 |
| BPAF | 2 | Brambling-Chinese juniper | 1 | 0.16 |
| BPAF | 2 | Japanese Tit-Chinese juniper | 1 | 0.16 |
| BPAF | 2 | Japanese Tit-Oriental arborvitae | 1 | 0.16 |
| BPAF | 2 | Red-Throated Thrush-Chinese juniper | 1 | 0.16 |
| BPAF | 2 | Tree Sparrow-Chinese juniper | 1 | 0.16 |
| BPAF | 4 | Red-Billed Blue Magpie-Amur honeysuckle | 1 | 0.16 |
| BPAF | 10 | Azure-Winged Magpie-Malus spp. | 24 | 3.88 |
| BPAF | 10 | Light-Vented Bulbul-Amur honeysuckle | 3 | 0.49 |
| BPAF | 10 | Azure-Winged Magpie-Amur honeysuckle | 2 | 0.32 |
| BPAF | 11 | Azure-Winged Magpie-Malus spp. | 100 | 16.18 |
| BPAF | 11 | Azure-Winged Magpie-Amur honeysuckle | 23 | 3.72 |
| BPAF | 11 | Light-Vented Bulbul-Amur honeysuckle | 8 | 1.29 |
| BPAF | 11 | Dusky Thrush-Malus spp. | 4 | 0.65 |
| BPAF | 11 | Azure-Winged Magpie-Oriental arborvitae | 2 | 0.32 |
| BPAF | 11 | Red-Billed Blue Magpie-Malus spp. | 1 | 0.16 |
| BPAF | 12 | Brambling-Chinese juniper | 13 | 2.10 |
| BPAF | 12 | Azure-Winged Magpie-Chinese juniper | 11 | 1.78 |
| BPAF | 12 | Hawfinch-Chinese juniper | 6 | 0.97 |
| BPAF | 12 | Red-Throated Thrush-Chinese juniper | 3 | 0.49 |
| BPAF | 12 | Oriental Magpie-Chinese juniper | 1 | 0.16 |
| BPAF | 12 | Oriental Turtle Dove-Chinese juniper | 1 | 0.16 |
| THU | 1 | Azure-Winged Magpie-Loose-flowered Euonymus | 3 | 0.49 |
| THU | 1 | Azure-Winged Magpie-Chinese ash | 2 | 0.32 |
| THU | 1 | Azure-Winged Magpie-Date-plum | 1 | 0.16 |
| THU | 1 | Azure-Winged Magpie-Manitoba maple | 1 | 0.16 |
| THU | 1 | Naumann's Thrush-Chinese juniper | 1 | 0.16 |
| THU | 1 | Oriental Magpie-Date-plum | 1 | 0.16 |
| THU | 1 | Red-Throated Thrush-Date-plum | 1 | 0.16 |
| THU | 2 | Azure-Winged Magpie-Chinese ash | 8 | 1.29 |
| THU | 2 | Azure-Winged Magpie-Rockspray cotoneaster | 1 | 0.16 |
| THU | 3 | Azure-Winged Magpie-Rockspray cotoneaster | 3 | 0.49 |
| THU | 6 | Oriental Magpie-Amur honeysuckle | 4 | 0.65 |
| THU | 6 | Spotted Dove-Amur honeysuckle | 2 | 0.32 |
| THU | 7 | Azure-Winged Magpie-Malus spp. | 1 | 0.16 |
| THU | 7 | Oriental Magpie-Amur honeysuckle | 1 | 0.16 |
| THU | 10 | Light-Vented Bulbul-Amur honeysuckle | 38 | 6.15 |
| THU | 10 | Azure-Winged Magpie-Malus spp. | 8 | 1.29 |
| THU | 10 | Azure-Winged Magpie-Amur honeysuckle | 5 | 0.81 |
| THU | 10 | Chinese Blackbird-Amur honeysuckle | 3 | 0.49 |
| THU | 10 | Azure-Winged Magpie-Rockspray cotoneaster | 1 | 0.16 |
| THU | 10 | Chinese Grosbeak-Malus spp. | 1 | 0.16 |
| THU | 10 | Light-Vented Bulbul-Malus spp. | 1 | 0.16 |
| THU | 10 | White Cheeked Starling-Rockspray cotoneaster | 1 | 0.16 |
| THU | 11 | Azure-Winged Magpie-Malus spp. | 4 | 0.65 |
| THU | 11 | Light-Vented Bulbul-Malus spp. | 3 | 0.49 |
| THU | 11 | Azure-Winged Magpie-Amur honeysuckle | 2 | 0.32 |
| THU | 11 | Chinese Blackbird-Rockspray cotoneaster | 1 | 0.16 |
| THU | 11 | Chinese Grosbeak-Malus spp. | 1 | 0.16 |
| THU | 11 | Light-Vented Bulbul-Amur honeysuckle | 1 | 0.16 |
| THU | 11 | Light-Vented Bulbul-Rockspray cotoneaster | 1 | 0.16 |
| THU | 11 | Marsh Tit-Rockspray cotoneaster | 1 | 0.16 |
| THU | 12 | Tree Sparrow-Date-plum | 9 | 1.46 |
| THU | 12 | Azure-Winged Magpie-Manitoba maple | 6 | 0.97 |
| THU | 12 | Light-Vented Bulbul-Date-plum | 5 | 0.81 |
| THU | 12 | Oriental Magpie-Manitoba maple | 4 | 0.65 |
| THU | 12 | Azure-Winged Magpie-Date-plum | 3 | 0.49 |
| THU | 12 | Azure-Winged Magpie-Chinese ash | 1 | 0.16 |

Table S9 Diurnal pattern of bird species and their abundance at the three sites

| Location | Season | Hour | Bird species | Abundance | Percentage |
| --- | --- | --- | --- | --- | --- |
| BAFLA | Spring | 5 | Azure-Winged Magpie | 1 | 0.07 |
| BAFLA | Spring | 8 | Azure-Winged Magpie | 1 | 0.07 |
| BAFLA | Spring | 11 | Azure-Winged Magpie | 1 | 0.07 |
| BAFLA | Spring | 14 | Azure-Winged Magpie | 1 | 0.07 |
| BAFLA | Spring | 16 | Azure-Winged Magpie | 1 | 0.07 |
| BAFLA | Summer | 5 | Azure-Winged Magpie | 2 | 0.15 |
| BAFLA | Summer | 15 | Azure-Winged Magpie | 1 | 0.07 |
| BAFLA | Summer | 16 | Azure-Winged Magpie | 1 | 0.07 |
| BAFLA | Summer | 18 | Azure-Winged Magpie | 2 | 0.15 |
| BAFLA | Summer | 19 | Azure-Winged Magpie | 1 | 0.07 |
| BAFLA | Autumn | 7 | Hawfinch | 24 | 1.78 |
| BAFLA | Autumn | 7 | Brambling | 19 | 1.41 |
| BAFLA | Autumn | 7 | Chinese Blackbird | 2 | 0.15 |
| BAFLA | Autumn | 7 | Light-Vented Bulbul | 1 | 0.07 |
| BAFLA | Autumn | 8 | Hawfinch | 27 | 2.00 |
| BAFLA | Autumn | 8 | Brambling | 10 | 0.74 |
| BAFLA | Autumn | 8 | Azure-Winged Magpie | 2 | 0.15 |
| BAFLA | Autumn | 8 | Chinese Blackbird | 1 | 0.07 |
| BAFLA | Autumn | 8 | Light-Vented Bulbul | 1 | 0.07 |
| BAFLA | Autumn | 9 | Hawfinch | 3 | 0.22 |
| BAFLA | Autumn | 9 | Chinese Blackbird | 2 | 0.15 |
| BAFLA | Autumn | 10 | Hawfinch | 25 | 1.85 |
| BAFLA | Autumn | 10 | Brambling | 6 | 0.44 |
| BAFLA | Autumn | 10 | Azure-Winged Magpie | 1 | 0.07 |
| BAFLA | Autumn | 11 | Brambling | 25 | 1.85 |
| BAFLA | Autumn | 11 | Hawfinch | 19 | 1.41 |
| BAFLA | Autumn | 11 | Chinese Blackbird | 3 | 0.22 |
| BAFLA | Autumn | 11 | Azure-Winged Magpie | 2 | 0.15 |
| BAFLA | Autumn | 11 | Naumann's Thrush | 1 | 0.07 |
| BAFLA | Autumn | 12 | Brambling | 19 | 1.41 |
| BAFLA | Autumn | 12 | Hawfinch | 17 | 1.26 |
| BAFLA | Autumn | 12 | Chinese Blackbird | 3 | 0.22 |
| BAFLA | Autumn | 12 | Azure-Winged Magpie | 2 | 0.15 |
| BAFLA | Autumn | 13 | Hawfinch | 28 | 2.08 |
| BAFLA | Autumn | 13 | Brambling | 18 | 1.33 |
| BAFLA | Autumn | 13 | Azure-Winged Magpie | 2 | 0.15 |
| BAFLA | Autumn | 13 | Chinese Blackbird | 2 | 0.15 |
| BAFLA | Autumn | 14 | Hawfinch | 29 | 2.15 |
| BAFLA | Autumn | 14 | Brambling | 12 | 0.89 |
| BAFLA | Autumn | 14 | Azure-Winged Magpie | 5 | 0.37 |
| BAFLA | Autumn | 14 | Chinese Blackbird | 3 | 0.22 |
| BAFLA | Autumn | 15 | Hawfinch | 36 | 2.67 |
| BAFLA | Autumn | 15 | Brambling | 10 | 0.74 |
| BAFLA | Autumn | 15 | Azure-Winged Magpie | 3 | 0.22 |
| BAFLA | Autumn | 15 | Spotted Dove | 3 | 0.22 |
| BAFLA | Autumn | 16 | Brambling | 17 | 1.26 |
| BAFLA | Autumn | 16 | Azure-Winged Magpie | 2 | 0.15 |
| BAFLA | Autumn | 16 | Hawfinch | 2 | 0.15 |
| BAFLA | Autumn | 16 | Marsh Tit | 1 | 0.07 |
| BAFLA | Winter | 7 | Brambling | 5 | 0.37 |
| BAFLA | Winter | 7 | Hawfinch | 2 | 0.15 |
| BAFLA | Winter | 7 | Naumann's Thrush | 2 | 0.15 |
| BAFLA | Winter | 7 | Red-Throated Thrush | 2 | 0.15 |
| BAFLA | Winter | 8 | Brambling | 5 | 0.37 |
| BAFLA | Winter | 8 | Hawfinch | 3 | 0.22 |
| BAFLA | Winter | 8 | Red-Billed Blue Magpie | 1 | 0.07 |
| BAFLA | Winter | 9 | Azure-Winged Magpie | 4 | 0.30 |
| BAFLA | Winter | 9 | Hawfinch | 4 | 0.30 |
| BAFLA | Winter | 9 | Brambling | 1 | 0.07 |
| BAFLA | Winter | 9 | Marsh Tit | 1 | 0.07 |
| BAFLA | Winter | 9 | Naumann's Thrush | 1 | 0.07 |
| BAFLA | Winter | 9 | Red-Billed Blue Magpie | 1 | 0.07 |
| BAFLA | Winter | 9 | Red-Throated Thrush | 1 | 0.07 |
| BAFLA | Winter | 9 | Spotted Dove | 1 | 0.07 |
| BAFLA | Winter | 9 | White Cheeked Starling | 1 | 0.07 |
| BAFLA | Winter | 10 | Brambling | 19 | 1.41 |
| BAFLA | Winter | 10 | Hawfinch | 2 | 0.15 |
| BAFLA | Winter | 10 | Light-Vented Bulbul | 2 | 0.15 |
| BAFLA | Winter | 10 | Azure-Winged Magpie | 1 | 0.07 |
| BAFLA | Winter | 11 | Azure-Winged Magpie | 4 | 0.30 |
| BAFLA | Winter | 11 | Naumann's Thrush | 3 | 0.22 |
| BAFLA | Winter | 12 | Brambling | 3 | 0.22 |
| BAFLA | Winter | 12 | Azure-Winged Magpie | 2 | 0.15 |
| BAFLA | Winter | 12 | Hawfinch | 1 | 0.07 |
| BAFLA | Winter | 12 | Light-Vented Bulbul | 1 | 0.07 |
| BAFLA | Winter | 12 | Marsh Tit | 1 | 0.07 |
| BAFLA | Winter | 12 | Tree Sparrow | 1 | 0.07 |
| BAFLA | Winter | 13 | Azure-Winged Magpie | 2 | 0.15 |
| BAFLA | Winter | 13 | Great Spotted Woodpecker | 1 | 0.07 |
| BAFLA | Winter | 13 | Hawfinch | 1 | 0.07 |
| BAFLA | Winter | 13 | Marsh Tit | 1 | 0.07 |
| BAFLA | Winter | 13 | Oriental Magpie | 1 | 0.07 |
| BAFLA | Winter | 14 | Brambling | 13 | 0.96 |
| BAFLA | Winter | 14 | Azure-Winged Magpie | 1 | 0.07 |
| BAFLA | Winter | 14 | Chinese Blackbird | 1 | 0.07 |
| BAFLA | Winter | 14 | Hawfinch | 1 | 0.07 |
| BAFLA | Winter | 14 | Light-Vented Bulbul | 1 | 0.07 |
| BAFLA | Winter | 14 | Spotted Dove | 1 | 0.07 |
| BAFLA | Winter | 15 | Brambling | 14 | 1.04 |
| BAFLA | Winter | 15 | Azure-Winged Magpie | 4 | 0.30 |
| BAFLA | Winter | 15 | Spotted Dove | 3 | 0.22 |
| BAFLA | Winter | 15 | White Cheeked Starling | 3 | 0.22 |
| BAFLA | Winter | 15 | Hawfinch | 2 | 0.15 |
| BAFLA | Winter | 15 | Red-Throated Thrush | 2 | 0.15 |
| BAFLA | Winter | 16 | Brambling | 9 | 0.67 |
| BAFLA | Winter | 16 | Azure-Winged Magpie | 3 | 0.22 |
| BAFLA | Winter | 16 | Hawfinch | 1 | 0.07 |
| BPAF | Spring | 6 | Red-Billed Blue Magpie | 1 | 0.07 |
| BPAF | Autumn | 6 | Azure-Winged Magpie | 7 | 0.52 |
| BPAF | Autumn | 7 | Azure-Winged Magpie | 7 | 0.52 |
| BPAF | Autumn | 8 | Azure-Winged Magpie | 5 | 0.37 |
| BPAF | Autumn | 9 | Azure-Winged Magpie | 65 | 4.82 |
| BPAF | Autumn | 10 | Azure-Winged Magpie | 118 | 8.75 |
| BPAF | Autumn | 10 | Light-Vented Bulbul | 1 | 0.07 |
| BPAF | Autumn | 11 | Azure-Winged Magpie | 41 | 3.04 |
| BPAF | Autumn | 11 | Light-Vented Bulbul | 3 | 0.22 |
| BPAF | Autumn | 12 | Azure-Winged Magpie | 30 | 2.22 |
| BPAF | Autumn | 12 | Light-Vented Bulbul | 3 | 0.22 |
| BPAF | Autumn | 12 | Dusky Thrush | 1 | 0.07 |
| BPAF | Autumn | 13 | Azure-Winged Magpie | 20 | 1.48 |
| BPAF | Autumn | 13 | Light-Vented Bulbul | 3 | 0.22 |
| BPAF | Autumn | 14 | Azure-Winged Magpie | 51 | 3.78 |
| BPAF | Autumn | 14 | Dusky Thrush | 4 | 0.30 |
| BPAF | Autumn | 14 | Light-Vented Bulbul | 3 | 0.22 |
| BPAF | Autumn | 15 | Azure-Winged Magpie | 39 | 2.89 |
| BPAF | Autumn | 15 | Red-Billed Blue Magpie | 1 | 0.07 |
| BPAF | Autumn | 16 | Azure-Winged Magpie | 19 | 1.41 |
| BPAF | Autumn | 17 | Azure-Winged Magpie | 2 | 0.15 |
| BPAF | Winter | 7 | Brambling | 3 | 0.22 |
| BPAF | Winter | 7 | Red-Throated Thrush | 1 | 0.07 |
| BPAF | Winter | 8 | Brambling | 17 | 1.26 |
| BPAF | Winter | 8 | Hawfinch | 2 | 0.15 |
| BPAF | Winter | 10 | Tree Sparrow | 7 | 0.52 |
| BPAF | Winter | 10 | Azure-Winged Magpie | 1 | 0.07 |
| BPAF | Winter | 10 | Hawfinch | 1 | 0.07 |
| BPAF | Winter | 10 | Red-Throated Thrush | 1 | 0.07 |
| BPAF | Winter | 10 | Yellow-Throated Bunting | 1 | 0.07 |
| BPAF | Winter | 11 | Azure-Winged Magpie | 25 | 1.85 |
| BPAF | Winter | 11 | Light-Vented Bulbul | 10 | 0.74 |
| BPAF | Winter | 11 | Red-Throated Thrush | 8 | 0.59 |
| BPAF | Winter | 11 | Japanese Tit | 3 | 0.22 |
| BPAF | Winter | 11 | Hawfinch | 1 | 0.07 |
| BPAF | Winter | 11 | Oriental Magpie | 1 | 0.07 |
| BPAF | Winter | 12 | Light-Vented Bulbul | 17 | 1.26 |
| BPAF | Winter | 12 | Azure-Winged Magpie | 5 | 0.37 |
| BPAF | Winter | 12 | Brambling | 4 | 0.30 |
| BPAF | Winter | 13 | Brambling | 15 | 1.11 |
| BPAF | Winter | 13 | Azure-Winged Magpie | 11 | 0.82 |
| BPAF | Winter | 13 | Light-Vented Bulbul | 5 | 0.37 |
| BPAF | Winter | 13 | Tree Sparrow | 5 | 0.37 |
| BPAF | Winter | 13 | Japanese Tit | 1 | 0.07 |
| BPAF | Winter | 13 | Oriental Turtle Dove | 1 | 0.07 |
| BPAF | Winter | 14 | Azure-Winged Magpie | 33 | 2.45 |
| BPAF | Winter | 14 | Brambling | 6 | 0.44 |
| BPAF | Winter | 14 | Japanese Tit | 2 | 0.15 |
| BPAF | Winter | 14 | Light-Vented Bulbul | 2 | 0.15 |
| BPAF | Winter | 14 | Tree Sparrow | 1 | 0.07 |
| BPAF | Winter | 15 | Hawfinch | 2 | 0.15 |
| BPAF | Winter | 15 | Red-Throated Thrush | 2 | 0.15 |
| BPAF | Winter | 15 | Japanese Tit | 1 | 0.07 |
| BPAF | Winter | 16 | Brambling | 12 | 0.89 |
| BPAF | Winter | 17 | Red-Throated Thrush | 1 | 0.07 |
| THU | Spring | 7 | Azure-Winged Magpie | 2 | 0.15 |
| THU | Spring | 17 | Azure-Winged Magpie | 1 | 0.07 |
| THU | Summer | 9 | Oriental Magpie | 1 | 0.07 |
| THU | Summer | 10 | Azure-Winged Magpie | 2 | 0.15 |
| THU | Summer | 10 | Oriental Magpie | 1 | 0.07 |
| THU | Summer | 11 | Oriental Magpie | 1 | 0.07 |
| THU | Summer | 15 | Oriental Magpie | 2 | 0.15 |
| THU | Summer | 15 | Spotted Dove | 2 | 0.15 |
| THU | Autumn | 6 | Azure-Winged Magpie | 1 | 0.07 |
| THU | Autumn | 7 | Azure-Winged Magpie | 1 | 0.07 |
| THU | Autumn | 7 | Chinese Blackbird | 1 | 0.07 |
| THU | Autumn | 7 | Light-Vented Bulbul | 1 | 0.07 |
| THU | Autumn | 8 | Azure-Winged Magpie | 3 | 0.22 |
| THU | Autumn | 8 | Light-Vented Bulbul | 3 | 0.22 |
| THU | Autumn | 8 | Chinese Grosbeak | 1 | 0.07 |
| THU | Autumn | 9 | Light-Vented Bulbul | 9 | 0.67 |
| THU | Autumn | 9 | Marsh Tit | 1 | 0.07 |
| THU | Autumn | 10 | Light-Vented Bulbul | 10 | 0.74 |
| THU | Autumn | 10 | Chinese Blackbird | 1 | 0.07 |
| THU | Autumn | 11 | Light-Vented Bulbul | 5 | 0.37 |
| THU | Autumn | 11 | Azure-Winged Magpie | 1 | 0.07 |
| THU | Autumn | 11 | Chinese Blackbird | 1 | 0.07 |
| THU | Autumn | 12 | Light-Vented Bulbul | 4 | 0.30 |
| THU | Autumn | 12 | Azure-Winged Magpie | 3 | 0.22 |
| THU | Autumn | 13 | Light-Vented Bulbul | 9 | 0.67 |
| THU | Autumn | 13 | Azure-Winged Magpie | 1 | 0.07 |
| THU | Autumn | 13 | Chinese Grosbeak | 1 | 0.07 |
| THU | Autumn | 14 | Light-Vented Bulbul | 3 | 0.22 |
| THU | Autumn | 14 | Azure-Winged Magpie | 1 | 0.07 |
| THU | Autumn | 15 | Azure-Winged Magpie | 4 | 0.30 |
| THU | Autumn | 15 | Chinese Blackbird | 1 | 0.07 |
| THU | Autumn | 15 | Light-Vented Bulbul | 1 | 0.07 |
| THU | Autumn | 15 | White Cheeked Starling | 1 | 0.07 |
| THU | Autumn | 16 | Azure-Winged Magpie | 8 | 0.59 |
| THU | Autumn | 16 | Light-Vented Bulbul | 1 | 0.07 |
| THU | Autumn | 17 | Azure-Winged Magpie | 1 | 0.07 |
| THU | Winter | 7 | Tree Sparrow | 9 | 0.67 |
| THU | Winter | 7 | Azure-Winged Magpie | 4 | 0.30 |
| THU | Winter | 7 | Light-Vented Bulbul | 3 | 0.22 |
| THU | Winter | 8 | Tree Sparrow | 7 | 0.52 |
| THU | Winter | 8 | Azure-Winged Magpie | 1 | 0.07 |
| THU | Winter | 8 | Light-Vented Bulbul | 1 | 0.07 |
| THU | Winter | 9 | Azure-Winged Magpie | 4 | 0.30 |
| THU | Winter | 9 | Tree Sparrow | 1 | 0.07 |
| THU | Winter | 10 | Azure-Winged Magpie | 2 | 0.15 |
| THU | Winter | 10 | Light-Vented Bulbul | 1 | 0.07 |
| THU | Winter | 10 | Red-Throated Thrush | 1 | 0.07 |
| THU | Winter | 10 | Tree Sparrow | 1 | 0.07 |
| THU | Winter | 11 | Oriental Magpie | 7 | 0.52 |
| THU | Winter | 11 | Azure-Winged Magpie | 1 | 0.07 |
| THU | Winter | 12 | Azure-Winged Magpie | 5 | 0.37 |
| THU | Winter | 12 | Oriental Magpie | 2 | 0.15 |
| THU | Winter | 12 | Naumann's Thrush | 1 | 0.07 |
| THU | Winter | 13 | Azure-Winged Magpie | 4 | 0.30 |
| THU | Winter | 13 | Tree Sparrow | 2 | 0.15 |
| THU | Winter | 13 | Light-Vented Bulbul | 1 | 0.07 |
| THU | Winter | 14 | Azure-Winged Magpie | 6 | 0.44 |
| THU | Winter | 14 | Light-Vented Bulbul | 1 | 0.07 |
| THU | Winter | 15 | Azure-Winged Magpie | 14 | 1.04 |
| THU | Winter | 15 | Oriental Magpie | 1 | 0.07 |
| THU | Winter | 15 | Tree Sparrow | 1 | 0.07 |
| THU | Winter | 16 | Tree Sparrow | 4 | 0.30 |
| THU | Winter | 16 | Azure-Winged Magpie | 2 | 0.15 |

Hawfinch’s foraging on Shantung maple trees at BAFLA accounted for 10.7% (66 times) of all frugivory events. At BPAF, Azure-winged Magpie and Malus spp. formed the dominant pairs, contributing to 20.1% (124 times) of all events. The interaction between Light-vented Bulbul and Amur honeysuckle at THU was ranked at the top, accounting for 6.3% (39 times) of all interactions. At BAFLA, the Azur-winged Magpie was the only species whose frugivory events were captured by the cameras in the spring and summer. Nevertheless, in the autumn and winter, other bird species such as Hawfinch and Brambling were more active than the Azur-winged Magpie at BAFLA.

As shown in Table S10, increasing the interval from 3 to 4 minutes reduced the number of events by 31 out of 618 (5.0%), and extending it to 5 minutes reduced the total by 52 (8.4%). These reductions are small compared with the higher event counts produced by shorter intervals (2 minutes: 650 events; 1 minute: 738 events), which would cause excessive splitting of continuous bouts. Therefore, 3 minutes represents the inflection point of the sensitivity curve and provides the most appropriate balance between maintaining independence and minimizing event loss.

Table S10. Sensitivity analysis of time gap thresholds on independent event identification.

| Time Gap | Independent Events | Reduction Rate |
| --- | --- | --- |
| 1 min | 738 | 73.6% |
| 2 min | 650 | 76.7% |
| 3 min | 618 | 77.9% |
| 4 min | 587 | 79.0% |
| 5 min | 566 | 79.7% |
| 10 min | 506 | 81.9% |
| 15 min | 465 | 83.4% |
| 30 min | 410 | 85.3% |

Note: “Reduction rate” is the percentage of raw detections that were merged when defining independent events under each time gap threshold.
